# A Photo‐degradable Crosslinker for the Development of Light‐responsive Protocell Membranes

**DOI:** 10.1002/chem.202302058

**Published:** 2023-09-22

**Authors:** Patrick J. Grimes, Mary Jenkinson‐Finch, Henry E. Symons, Wuge H. Briscoe, Sebastien Rochat, Stephen Mann, Pierangelo Gobbo

**Affiliations:** ^1^ School of Chemistry University of Bristol Cantock's Close Bristol BS8 1TS UK; ^2^ School of Engineering Mathematics and Technology University of Bristol Ada Lovelace Building Tankard's Close Bristol BS8 1TW UK; ^3^ Department of Chemical and Pharmaceutical Sciences University of Trieste Via L. Giorgieri 1 Trieste 34127 Italy; ^4^ National Interuniversity Consortium of Materials Science and Technology Unit of Trieste Via G. Giusti 9 Firenze 50121 Italy

**Keywords:** nonequilibrium processes, photo-degradable crosslinker, photolysis, proteinosomes, protocell

## Abstract

The achievement of light‐responsive behaviours is an important target for protocell engineering to allow control of fundamental protocellular processes such as communication via diffusible chemical signals, shape changes or even motility at the flick of a switch. As a step towards this ambitious goal, here we describe the synthesis of a novel poly(ethylene glycol)‐based crosslinker, reactive towards nucleophiles, that effectively degrades with UV light (405 nm). We demonstrate its utility for the fabrication of the first protocell membranes capable of light‐induced disassembly, for the photo‐generation of patterns of protocells, and for the modulation of protocell membrane permeability. Overall, our results not only open up new avenues towards the engineering of spatially organised, communicating networks of protocells, and of micro‐compartmentalised systems for information storage and release, but also have important implications for other research fields such as drug delivery and soft materials chemistry.

## Graphical abstract

**A novel photocleavable, poly(ethylene) glycol‐based crosslinker**, reactive to biological nucleophiles is synthesized and used to covalently stabilise proteinosome membranes. The crosslinker effectively degrades on irradiation with UV light, allowing for the photopatterning of populations of proteinosomes using a 405 nm laser, and for the controlled formation of membrane pores and the consequent controlled release of macromolecular cargoes based on their molecular weight.

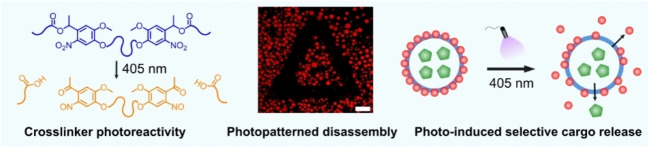

## Introduction

The development of protocell technology has advanced rapidly over the past decade, with significant progress in the areas of protocell communication, engineering and chemical programming of protocell membranes.^[1]^ These areas are of great interest as their development provides frameworks for the fabrication of out‐of‐equilibrium networks for microreactor technologies, biosensing and drug delivery. Moreover, this form of research provides a simplified environment to probe fundamental, physico‐chemical aspects of biology in a highly controlled manner.

**Figure 1:**
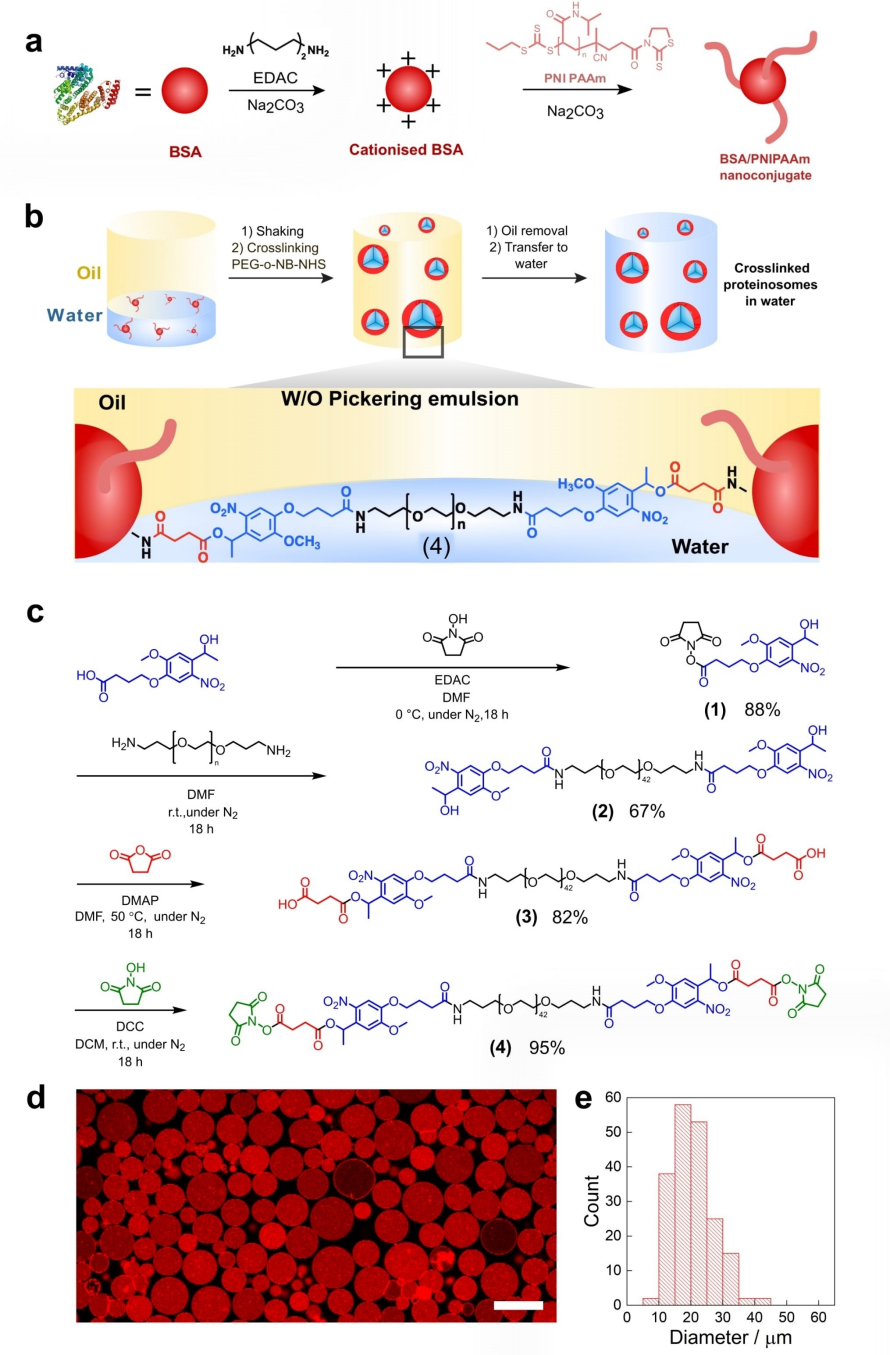
Fabrication of PEG‐*o*‐NB‐NHS crosslinked proteinosomes. a) Schematic of amphiphilic BSA/PNIPAAm nanoconjugate synthesis. Na_2_CO_3 (aq)_ used was 0.1 M and adjusted to pH 8.5. All synthetic details are reported in Supporting Information Sections 1.3–1.5. b) Synthesis of light‐responsive *ortho*‐nitrobenzyl (*o*‐NB) proteinosomes. BSA/PNIPAAm nanoconjugates in water are mixed with a solution of PEG‐*o*‐NB‐NHS in carbonate buffer (0.1 M, pH 8.5). A layer of oil is added on top, and the mixture vigorously shaken to form water‐in‐oil droplets, where BSA/PNIPAAm nanoconjugates stabilise the surface of the droplets, and PEG‐*o*‐NB‐NHS covalently joins them together by reacting with the residual free amine groups on BSA. All details of light‐responsive *o*‐NB proteinosome preparation are reported in Supporting Information Section 1.6. c) Synthetic route to photocleavable crosslinker (PEG)‐bis([*o*‐nitro benzyl] *N*‐succinimidyl succinate) (PEG‐*o‐*NB‐NHS) (**4**). d) Confocal laser scanning microscopy image of a population of RITC‐tagged *o*‐NB proteinosomes in water. Scale bar=25 μm. e) Size distribution of *o*‐NB proteinosomes showing an average diameter of 22.5±6.1 μm.

Amongst the plethora of different protocell models available, *proteinosomes* have garnered much attention due to their ability to entrap a wide variety of guest molecules, including those required for cell‐free gene expression.^[2]^ Proteinosomes are membrane‐bound microcapsules, composed of a self‐assembled monolayer of amphiphilic organic nanoparticles, covalently bonded together via a poly(ethylene glycol) (PEG)‐based crosslinker, terminated with amine‐reactive *N*‐hydroxysuccinimide (NHS) esters. Typically, in proteinosomes, the amphiphilic colloidal nanoparticle comprises a cationised protein core (bovine serum albumin, BSA), functionalised with up to 3 poly(*N*‐isopropylacrylamide) (PNIPAAm) chains (*ca*. 10 kDa each) (Figure 1a) and a fluorophore (e.g., fluorescein isothiocyanate (FITC) or rhodamine B isothiocyanate (RITC)) to enable its visualisation through fluorescence microscopy. These amphiphilic nanoparticles stabilise a water‐in‐oil Pickering emulsion, whilst the PEG‐based crosslinker covalently bonds the nanoparticles together by reacting with the free amine residues of BSA, forming a spherical semi‐permeable microcompartment (Figure 1b). The proteinosomes can then be transferred to water, retaining their colloidal membrane as a result of the covalent bonds formed between the PEG crosslinkers and the protein‐polymer nanoconjugates. Without the crosslinker, the proteinosomes would disassemble on transfer to bulk aqueous solution. Due to the physico‐chemical properties of PNIPAAm, proteinosomes are also inherently thermoresponsive, and their membranes can be functionalised with bio‐orthogonal functional groups to enable their assembly into free‐standing protocellular materials.^[3]^

We have recently been investigating the use of external stimuli, such as light or pH, to trigger higher order protocell behaviours such as membrane adhesion, total membrane disassembly and protocell capture.[^1c^, ^4^] In particular, photoresponsive properties in synthetic cells are of great interest because of their direct relevance to fields such as drug delivery and synthetic cell signalling. The use of light as an external stimulus has many advantages including precise modulation of input intensity and excellent spatiotemporal control. Furthermore, light is used widely in nature as a stimulus and as an energy source for multiple biological processes, and therefore the use of light as an input in protocell systems is highly applicable to the field of bottom‐up synthetic biology. Indeed, several studies made use of the beneficial properties of light in protocell systems, for example, to initiate DNA strand displacement reactions for protocell communication or to provide energy for NADH production.^[5]^

Photocleaving or degrading moieties are widespread in chemical synthesis and materials chemistry and are increasingly being investigated in the context of drug delivery and synthetic biology.^[6]^ Commonly used photocleaving groups include nitroaryls,^[7]^ arylcarbonylmethyls,^[8]^ coumarin‐4‐ylmethyls,^[9]^ as well as Ru‐containing complexes.^[10]^ Of the multitude of photoresponsive moieties available for use in light‐responsive functional materials and protocells, the *ortho*‐nitrobenzyl (*o*‐NB) functionality has enjoyed widespread employment in a variety of chemical and living systems, including photoresponsive hydrogels and drug delivery systems, making this group ideal for use in bottom‐up synthetic biology.[^11^, ^12^] Following excitation of the *o‐*NB moiety with UV light, the nitro group is transformed to the reactive *aci*‐nitro functionality, which abstracts a proton from the benzylic carbon *ortho* to the nitro group, allowing an irreversible 5‐membered ring formation. Subsequent cleavage of this ring produces a carboxylic acid and an *o*‐nitrosobenzaldehyde as photoproducts (see Supporting Information Figure S1).^[13]^ The *o*‐NB moiety has a broad absorption peak at 350 nm (see Supporting Information Figure S2) but is susceptible to reaction across a broad range of wavelengths (from 350 to >400 nm).

Despite the ubiquitous use of *o*‐NB functionality in photoresponsive systems, to the best of our knowledge, a linear PEG‐based crosslinker which incorporates this mode of photoresponsivity and facile reaction with biologically relevant nucleophiles has not been reported.^[14]^

Here, we describe the synthesis of a novel PEG‐based crosslinker with incorporated *o*‐NB functionality. We demonstrate the use of this novel crosslinker to synthesise populations of proteinosomes with nanometre‐thick membranes that cleave in a controlled manner following exposure to UV radiation.^[2a]^ This behaviour was exploited to pattern protocell populations and to control the release of molecular cargoes based on their molecular weight, as a step towards the engineering of more complex forms of protocell‐protocell or protocell‐living cell communication based on diffusible chemical signals.

From a general perspective, our results describe a novel route towards photoresponsive protocells and a new method to control the release of chemical information from inside protocell lumina through modification of membrane porosity. This methodology will provide new opportunities for potential applications of proteinosomes in protocell research, soft matter chemistry and drug delivery.

## Results and Discussion

### Synthesis of PEG‐o‐NB‐NHS

Synthesis of the *o*‐NB crosslinker (Figure 1c; Section 1.2, Supporting Information) was achieved in four steps from commercial starting materials, beginning with the activation of the carboxylic acid group on 4‐[4‐(1‐hydroxyethyl)‐2‐methoxy‐5‐nitrophenoxy]butyric acid (*o*‐NB) with 1.5 molar equivalents of *N*‐hydroxysuccinimide (NHS) via a 1‐ethyl‐3‐(3 dimethylaminopropyl)carbodiimide‐ (EDAC) ‐mediated amide coupling to afford compound **(1)** in 88 % yield. Reaction of **(1)** with a bis‐amine terminated poly(ethylene glycol) (PEG) chain (*M*_n_=1500 g mol^−1^) gave **(2)** in 67 % yield. A change in the terminal functional groups of **(2)** from secondary alcohols to carboxylic acids was required to activate the final crosslinker product with NHS groups. This transformation was achieved via the base‐mediated nucleophilic addition of the alcoholic termini of **(2)** to succinic anhydride producing compound **(3)** in 82 % yield. The crosslinker synthesis was completed with the addition of NHS groups (via a dicyclohexylcarbodiimide‐mediated amide coupling reaction) to the terminal carboxylic acids of **(3)** to form electrophilic NHS esters (PEG‐*o*‐NB‐NHS **(4)**, 95 %). All intermediate and final products were characterised via ^1^H NMR, ^13^C NMR, matrix‐assisted laser desorption ionisation – time of flight (MALDI‐TOF) mass spectrometry, FTIR spectroscopy, UV‐Vis spectroscopy and gel permeation chromatography (GPC) (Supporting Information Figures S3–S19). In particular, ^1^H NMR spectroscopy of the first intermediate (PEG‐*o*‐NB, **(2)**) and the final crosslinker (PEG‐*o‐*NB‐NHS, **(4)**) showed that the conjugation of the *o*‐NB group to the main PEG backbone was stoichiometric, as well as the excellent formation of electrophilic NHS esters, respectively. Diagnostic peaks at 5.46 and 3.6 ppm (intermediate **(2)**, Supporting Information Figure S5) verified the ratio of *o*‐NB protons to PEG backbone protons. A diagnostic peak in the ^1^H NMR spectrum of **(4)** at 2.88 ppm confirmed the successful formation of NHS esters (Supporting Information Figure S16). MALDI‐TOF mass spectrometry and GPC data showed that **(4)** did not decompose under the reaction conditions used and that the mass of the polymer increased due to the progressive modification of the end groups as described in Figure 1(c).

The crosslinker product was stable for several months when stored in darkness under an atmosphere of nitrogen at −18 °C and was used directly, without further purification. Kinetics data of UV‐induced photocleavage (Supporting Information Figures S20 and S21) were obtained by irradiating a 0.1 mM solution of intermediate **(3)** in phosphate‐buffered solution (0.1 M, pH 7.4) with a 365 nm xenon light source (11 mW). Using UV‐Vis spectroscopy, we found that the photocleavage reaction was complete after approximately 20 min of irradiation, exhibiting first order reaction kinetics, in agreement with examples in the literature.[^13a^, ^15^] Taken together, these data confirm successful synthesis of the photo‐cleavable crosslinker and its effective photoreactivity.

### Photo‐induced disassembly of o‐NB proteinosomes

Using our novel photocleavable crosslinker, PEG‐*o*‐NB‐NHS **(4)**, we synthesised a population of light‐sensitive proteinosome*s* using a method adapted from our previously reported work.^[2a]^ First, we synthesised the protein‐polymer nanoconjugate (Supporting Information Sections 1.3–1.5). Using readily available bovine serum albumin (BSA) for the protein core of the nanoconjugate, we first increased the number of primary amine residues on the surface of BSA by coupling hexamethylenediamine to its interfacial carboxylic acid residues (achieved using an EDAC‐mediated amide coupling reaction) to yield cationised BSA (BSA‐NH_2_) (Figure 1a). Through MALDI‐TOF mass spectrometry, we determined that the degree of BSA cationisation was 28.8 % – i. e., given that BSA has 100 aspartate and glutamate residues, we successfully transformed approximately 29 of the BSA carboxylate residues into primary amine groups (Supporting Information Figure S22). Next, BSA‐NH_2_ was tagged with rhodamine B‐ or fluorescein‐isothiocyanate (RITC or FITC, respectively) to allow for the visualisation of the protein‐polymer nanoconjugate and of the final proteinosomes through fluorescence microscopy. UV‐Vis spectroscopy was used to calculate the degree of fluorescent labelling of BSA‐NH_2_ as 20 %, meaning that only a small fraction of the total protein molecules were tagged with a fluorophore and, consequently, that many free amine groups remained available on the protein surface for further reaction (Supporting Information Figure S23). Next, a mercaptothiazoline‐terminated PNIPAAm polymer (*M*_n_ 8,745 Da; *M*_w_ 9,620 Da; *Đ*_M_ 1.1) was synthesised via reversible addition‐fragmentation chain transfer (RAFT) polymerisation according to our previously reported procedure.^[2a]^ The amine‐reactive PNIPAAm polymer was grafted on to the tagged BSA‐NH_2_; at least 3 PNIPAAm chains were covalently bonded to each BSA‐NH_2_ molecule (based on MALDI‐TOF data, Supporting Information Figure S24). Adding PNIPAAm chains to BSA‐NH_2_ increased the lipophilicity of the cationised protein, allowing it to stabilise water‐in‐oil emulsions effectively. To synthesise *o‐*NB proteinosomes, a solution of fluorescently tagged BSA/PNIPAAm nanoconjugates in MilliQ water (30 μL, 8 mg mL^−1^) was mixed with a solution of *o*‐NB crosslinker **(4)** (30 μL, 67 mg mL^−1^) in sodium carbonate solution (pH 8.5, 100 mM) and a layer of 2‐ethyl‐1‐hexanol (1 mL) was added on top of the aqueous phase (water/oil volume ratio of 0.06). A water‐in‐oil emulsion was generated by manually shaking the mixture vigorously for 30 seconds (Figure 1b, Supporting Information Section 1.6). After 48 h of incubation in the absence of light, the covalently crosslinked proteinosomes were transferred to water via dialysis against a decreasing concentration of ethanol in water (70 % to 0 % over 24 h). The successful transfer to water confirmed the effective formation of covalently crosslinked proteinosomes. After transfer, the proteinosomes were kept for 48 h in darkness at 5 °C to allow for their sedimentation, then were washed and re‐sedimented 3 times to remove any membrane fragments. A confocal laser scanning microscope (CLSM) was used to characterise the proteinosomes. Figure 1(d, e) shows a population of spherical, covalently crosslinked proteinosomes with an average diameter of 22.5±6.1 μm; the proteinosomes were stable for months when stored in darkness at 5 °C. In general, the proteinosome membrane was not well defined in the CLSM images due to the high red fluorescence of the proteinosome lumina associated with the encapsulation of excess RITC‐tagged BSA/PNIPAAm nanoconjugates during protocell fabrication. These conditions were employed to increase the signal‐to‐noise ratio in the semi‐quantitative data analysis of membrane photo‐degradation.

**Figure 2:**
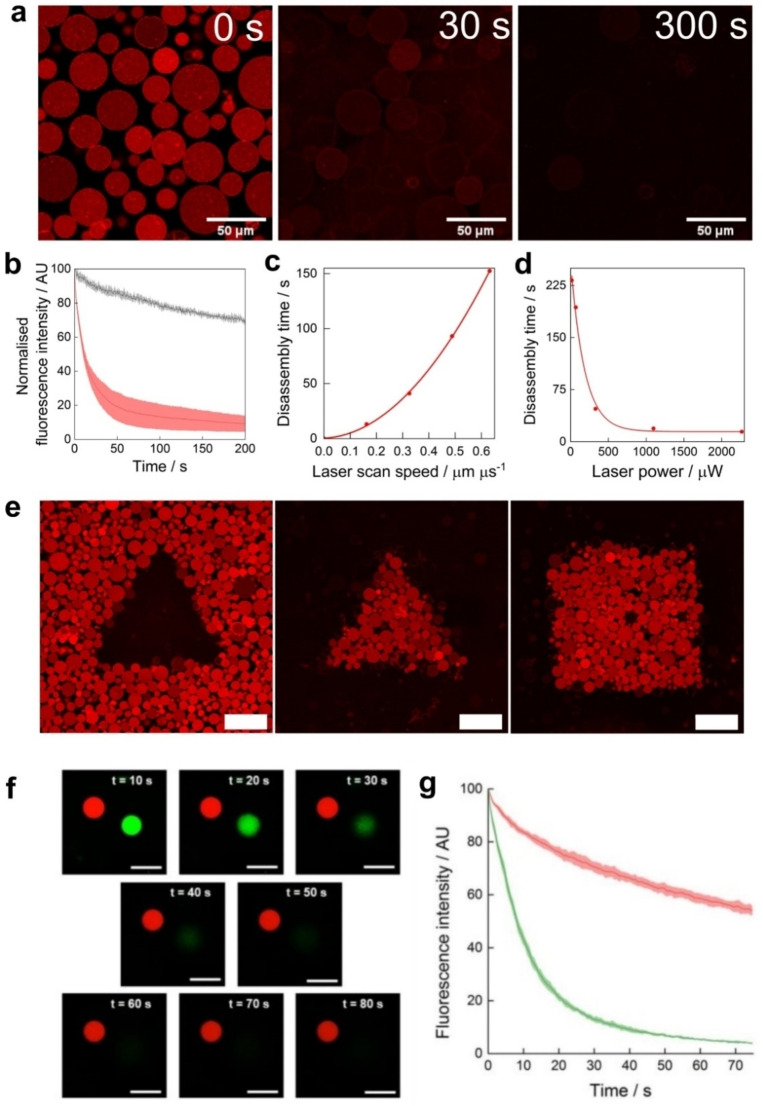
Study of the photochemical behaviour of *o*‐NB proteinosomes. a) Time‐dependent CLSM images of a population of *o*‐NB proteinosomes disassembling over a period of 300 seconds, during exposure to a 405 nm laser (50 mW). b) Plot of fluorescence intensity vs. time for a population of photo‐sensitive *o*‐NB proteinosomes (red trace) and photo‐stable PEG‐diNHS proteinosomes (control experiment, grey trace) during 200 s of irradiation with a 405 nm laser. Error bands: standard deviation. c) Plot of proteinosome disassembly time as a function of laser scan speed. The laser scan speed was systematically varied between 0.16 and 0.63 μm ms^−1^ and the corresponding disassembly time increased exponentially from 13 to 152 s, respectively. d) Plot of proteinosome disassembly time as a function of laser power. The laser power was systematically varied between 17 and 2266 mW and the corresponding disassembly time decreased exponentially from 232 to 14 s. e) CLSM images of photo‐generated patterns in a population of RITC‐tagged *o*‐NB proteinosomes. The populations were irradiated for 5 min with 405 nm laser light. Scale bars=100 μm. f) CLSM images of a photo‐sensitive, FITC‐tagged, PEG‐*o*‐NB‐NHS crosslinked proteinosome (green) and a photo‐stable, RITC‐tagged, PEGdiNHS crosslinked proteinosome (red). Both proteinosomes were simultaneously irradiated with a 405 nm laser (50 mW), inducing photolysis only in the green proteinosome. Scale bars=25 μm. g) Time‐dependent fluorescence intensity plot for both the photo‐sensitive, FITC‐tagged PEG‐*o*‐NB‐NHS crosslinked proteinosome (green plot) and the photo‐stable, RITC‐ tagged PEG‐diNHS crosslinked proteinosome (red plot) shown in (f). The green curve shows a rapid decrease of fluorescence intensity over time due to the photolysis of the photo‐sensitive proteinosome membrane. The red curve shows instead a much slower monotonic decrease of fluorescence intensity due to photobleaching of RITC. Error bands: standard deviation.

In order to showcase the ability of our *o*‐NB proteinosomes to disassemble on irradiation with UV light, we employed CLSM to irradiate a proteinosome population enclosed in an area of 388 μm x 388 μm with a 50 mW, 405 nm laser (Figure 2a, Supporting Information Section 1.7, Supporting Video **1**). CLSM images of the population taken at 0 s, 30 s and 300 s showed a reduction of almost 100 % in the average fluorescence intensity of the population following its continual irradiation with the 405 nm light (Figure 2b, red plot). We attributed this fluorescence intensity decrease to cleavage of the *o*‐NB‐functionalised crosslinker, membrane disassembly, and subsequent diffusion and dilution of the fluorescent BSA‐PNIPAAm nanoconjugates into the surrounding bulk aqueous solution. In contrast, a control experiment carried out with photostable PEG‐diNHS crosslinked proteinosomes only showed a 20 % decrease in the fluorescence intensity (Figure 2b grey plot, Supporting Information Figure S25), which was attributed to the photobleaching of the RITC tag under the experimental conditions. These results indicated that the decrease in fluorescence intensity of the proteinosomes crosslinked with PEG‐*o*‐NB‐NHS was mainly due to the cleavage of the *o*‐NB moiety present in the crosslinker, and not due to photobleaching of the fluorescently tagged BSA/PNIPAAm nanoconjugate. To quantify the rate of proteinosome photo‐degradation, we defined a “proteinosome disassembly time” – the time point at which the linear regression of the initial fluorescence intensity decay and that of the plateau region of fluorescence intensity intersected (Supporting Information Figure S26). We observed that the proteinosome disassembly time could be carefully controlled through manipulation of the confocal laser scan speed and power respectively, and could be systematically varied from 13 to 152 s (low to high laser scan speed) and from 232 to 14 s (low to high laser power) (Figure 2c and d; Supporting Information Sections 1.8 and 1.9, Figures S27 and S28).

In order to study the photolysis behaviour of a large population of *o*‐NB proteinosomes, fluorescence‐activated cell sorting (FACS) was used (Supporting Information Figure S29, Section 1.10). In a vial, a population of PEG‐*o*‐NB‐NHS‐crosslinked, FITC‐labelled proteinosomes was exposed to prolonged irradiation with a 365 nm xenon light source (11 mW). Supporting Information Figure S29 shows a typical two‐dimensional plot of fluorescence intensity (FITC−H) vs. side light scattered area (SSC−A) with a distinguishable population with high fluorescence, which, based on our previous experience,^[1c]^ was attributed to proteinosomes.

The same plot at *t*=0 min also shows a side scattering signal, which was instead attributed to broken proteinosomes that inevitably formed when transferred from a water‐in‐oil system to a bulk aqueous phase. After 9 min of irradiation, the signal from the proteinosome population decreased from 91.4 % to 7.8 % and after 18 min it reduced further to 1.8 %. This was accompanied by a progressive increase in the membrane fragment population, indicative of an effective disassembly of the proteinosomes.

Next, we showed that patterns could be photo‐generated in a population of *o*‐NB proteinosomes (Figure 2e, Supporting Information Section 1.11). To achieve this, a sample of light‐sensitive proteinosomes in MilliQ water was allowed to settle in a channel slide, forming a monolayer of protocells. The CLSM software allowed spatially defined regions of interest (ROIs) to be irradiated with a 405 nm UV laser, such that bespoke patterns of proteinosomes of any shape in the 100 μm scale could be photo‐generated. Within the user‐defined ROIs, UV‐illumination caused photo‐degradation of the proteinosomes, forming negative shapes in the proteinosome monolayer. This technique was also exploited to leave positive shapes by irradiating the regions around the desired pattern, and a combination of positive and negative shape generation allowed more complex patterns to be created (Supporting Information Figure S30).

Moreover, we showed that within a binary population of proteinosomes, the photo‐cleavable crosslinker allowed us to selectively disassemble one population over the other. To demonstrate this selectivity, we placed a binary population of proteinosomes in a Petri dish, comprising 50 % FITC‐tagged proteinosomes crosslinked with PEG‐*o*‐NB‐NHS, and 50 % RITC‐tagged proteinosomes crosslinked with photo‐stable PEG‐diNHS. Irradiation of the binary population of proteinosomes caused the selective disassembly of the FITC‐tagged proteinosomes, as shown by the CLSM time‐lapse in Figure 2(f) and by the time‐dependent fluorescence intensity analysis reported in Figure 2(g) (Supporting Information Videos 2 and 3).

Taken together, these results not only demonstrate the efficacy of our photo‐degradable crosslinker in proteinosome photolysis, but also showcase the high degree of spatiotemporal control available for the patterning of proteinosome populations, as well as the ability to selectively target and degrade a specific proteinosome population over another.

### Controlled release of molecular cargoes from proteinosomes based on molecular weight

As described above, we have produced proteinosomes with membranes that can be disassembled in a highly controlled fashion on irradiation with UV light. In order to make use of this new methodology, we attempted to entrap and then release high molecular weight cargoes from within the proteinosome lumen as models for signaling molecules. To achieve this, a thorough understanding of the proteinosome membrane molecular weight cut‐off (MWCO) was required. Therefore, we synthesised populations of PEG‐*o*‐NB‐NHS crosslinked proteinosomes following our standard method, but also including 6 mg mL^−1^ solutions of FITC‐tagged dextran with different molecular weights (40, 70, 150 and 2,000 kDa, see Supporting Information Section 1.12) in the aqueous phase of the proteinosome emulsion. Using CLSM, we determined the levels of FITC‐dextran retained inside the proteinosome lumina after transfer to bulk aqueous solution by calculating the ratio between the intensity of green fluorescence inside the proteinosome (*FI*_inside_) to the intensity of green fluorescence outside the proteinosome (*FI*_outside_). These experiments suggested a MWCO of between 40 and 70 kDa (Supporting Information Figure S31) with an *FI*_inside_ /*FI*_outside_ ratio of *ca*. 1 for the 40 kDa FITC‐dextran and a ratio of *ca*. 7 for the 70 kDa FITC‐dextran. These results are in line with those previously published by our group and colleagues,[^3a^, ^4b^] and were further confirmed by developing a physicochemical model that described proteinosome membrane permeability, which is reported in detail in the Supporting Information, Section 2.

**Figure 3:**
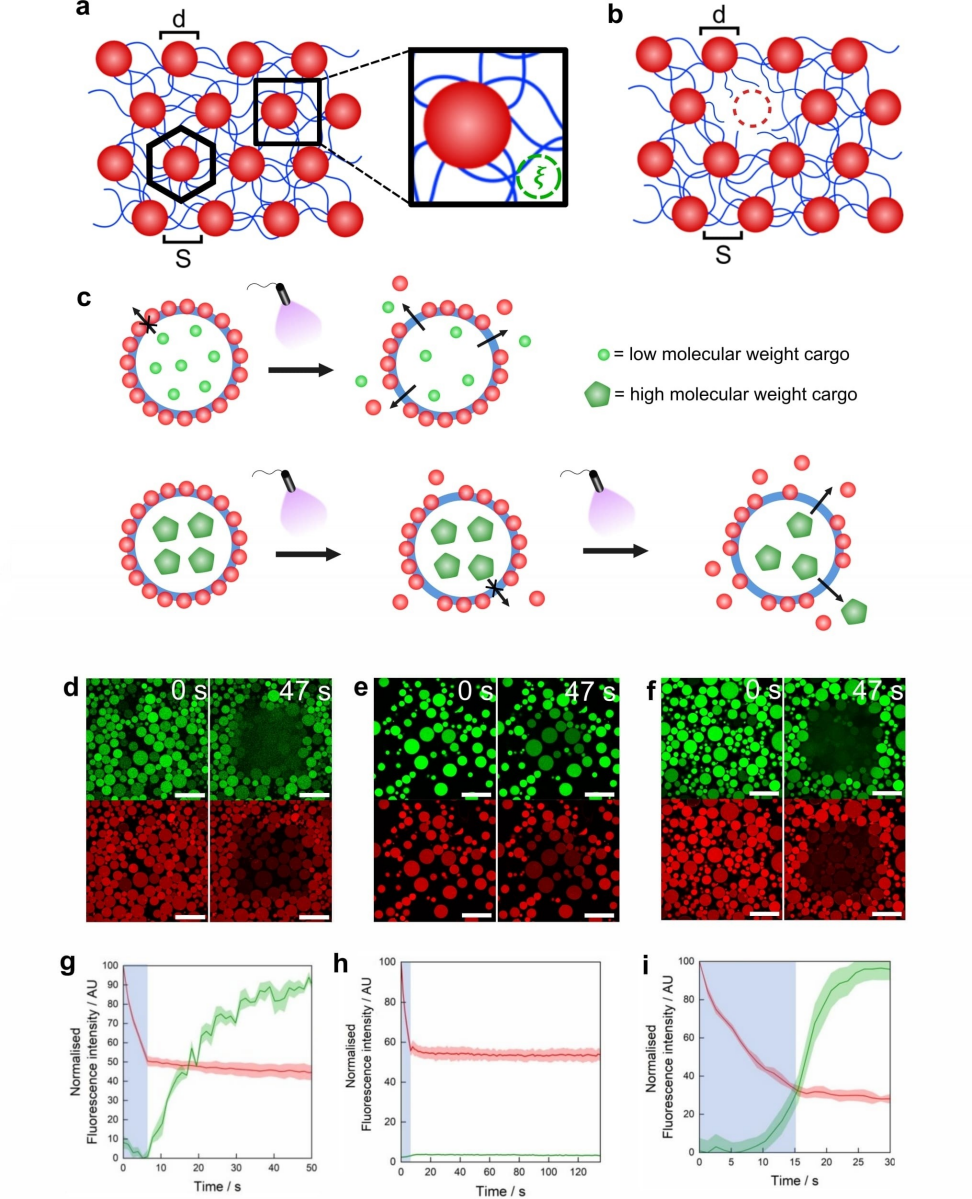
Study and light‐induced modification of *o*‐NB proteinosome membrane porosity. a) Scheme showing the proposed model of the proteinosome membrane comprising BSA/PNIPAAm nanoconjugates (red spheres of diameter *d*) in a PEG‐*o*‐NB‐NHS crosslinker network (blue lines) characterised by a mesh size *ξ*. It is assumed that the nanoconjugates are arranged in a hexagonal packing (a single hexagonal packing unit is schematically represented with a black hexagon) with hexagonal diameter (*d*+*S*) where *S* is the inter‐conjugate distance with a lower limit of ~5.6 nm (set by the hydrodynamic radius of the smallest 40 kDa FITC‐dextran that can diffuse though the mesh) and an upper limit of ~15 nm (set by the 150 kDa polymer size), as well as the contour length of the polymer. b) Scheme showing the proposed theoretical model of the proteinosome membrane as in (a), but showing the loss of one protein‐polymer nanoconjugate and the generated pore with a diameter ~*d*. The lost nanoconjugate and resultant pore is represented with a red dashed circle. c) Schematic diagram illustrating the selective release of a molecular cargo based on molecular weight. Red spheres represent nanoconjugates, blue circle represents the aqueous shell crosslinker network. d) CLSM images showing RITC‐tagged, PEG‐*o*‐NB‐NHS crosslinked proteinosomes loaded with 150 kDa FITC‐tagged dextran at 0 s and 47 s after 5.5 s irradiation with 405 nm laser (50 mW). Top images show FITC channel and bottom images show RITC channel. e) CLSM images showing RITC‐tagged, PEG‐*o*‐NB‐NHS crosslinked proteinosomes loaded with 2,000 kDa FITC‐tagged dextran at 0 s and 47 s after 5.5 s irradiation with 405 nm laser (50 mW). Top images show FITC channel and bottom images show RITC channel. f) CLSM images showing RITC‐tagged, PEG‐*o*‐NB‐NHS crosslinked proteinosomes loaded with 2,000 kDa FITC‐tagged dextran at 0 s and 47 s after 15.5 s irradiation with 405 nm laser (50 mW). Top images show FITC channel and bottom images show RITC channel. g–i) Normalised fluorescence intensity plot monitored during and after irradiation with a 405 nm laser (50 mW) for either 5.5 (g, h) or 15.5 s (i) (blue vertical bands). The green traces correspond to the intensity of the FITC fluorescent signal detected from regions immediately outside the proteinosome membranes. This signal is associated with the release (g, i) or retention (h) of the green fluorescent molecular cargo. The red traces correspond to the RITC fluorescent signal of proteinosome membranes. Graphs (g), (h), (i) correspond to the experiments in (d), (e), (f), respectively. Error bands: standard deviation.

In summary, the proteinosome membrane cannot be considered simply as a monolayer of hexagonally close packed BSA/PNIPAAm nanoconjugates, as these nanoparticles are covalently bound to a network of PEG crosslinker (Figure 3a). By applying the de Gennes theory,^[16]^ and therefore considering the crosslinked polymers of the proteinosome membrane as existing in the “semi‐dilute regime”, we could define a “mesh size”, *ξ* (inset, Figure 3a), which is comparable to the size of molecules that can freely diffuse through the proteinosome membrane. According to de Gennes, the mesh size for a semi‐dilute solution of polymers is given by Equation 1:

(1) $$\xi \;=a\varphi^{-3\;/\;\;4}$$

where *a* is the monomer length of PEG (0.35 nm),^[16]^ and *φ* is the polymer volume fraction which, in the case of proteinosomes, is dependent on the number of protein‐polymer nanoconjugates per proteinosome (*n*), the number of reactive free amine groups per BSA/PNIPAAm nanoconjugate (*z*), the molecular volume of all the crosslinkers in the membrane (*V_x_*, estimated to be between 6.3×10^9^ and 1.02×10^10^ nm^3^), and the volume pervaded by those crosslinkers (*V_L_*, estimated to be 2.74 nm^3^) as described in Equation 2.

(2) $$\varphi =\;\frac{\left(\frac{\mathrm{nz}}{4}\right)v_{L}}{V_{x}}$$

Values for *n=*2.10×10^6^ and *z=*85 were estimated following the methods described in Supporting Information Section 2. Using these estimates we obtained values for *φ* of between 0.012 and 0.050. Using the literature value for PEG monomer length (0.35 nm)^**[17]**^ and Equation (1), we calculated a mesh size for the proteinosome membrane of *ξ=*3.3–10.0 nm. This aligns remarkably well with our experimental results; the proteinosome membrane was able to effectively encapsulate 70 kDa dextran (hydrodynamic radius=12.3 nm) but was fully permeable to 40 kDa dextran (hydrodynamic radius=5.6 nm). With a precise determination of proteinosome membrane MWCO, we attempted to selectively release cargoes of different molecular weights. We proposed that pores could be generated during crosslinker photocleavage in the membrane, as individual nanoconjugates were released following the breakage of their crosslinker tethers. We hypothesised that by varying the incident UV radiation on the proteinosome population, we could tune the extent of membrane disassembly, and thus the pore size. Through control of membrane pore size, we could achieve selective release of different molecular weight cargoes based on their hydrodynamic radius (Figure 3a–c). For this, we synthesised two populations of RITC‐tagged proteinosomes crosslinked with PEG‐*o*‐NB‐NHS. The first population was loaded with 150 kDa FITC‐dextran and the second was loaded with 2,000 kDa FITC‐dextran (Supporting Information Section 1.13), with corresponding hydrodynamic radii of 16.5±1.2 nm and 20.1±1.4 nm, respectively (concentrations of FITC‐dextrans in proteinosome lumina=1 mg mL^−1^). When imaged under confocal microscopy (using multichannel imaging), the populations possessed red membranes with green lumina corresponding to RITC and FITC fluorescence output, respectively (see Figures 3d–f). Initially, a central square region (200 μm×200 μm) of the frame (338 μm×338 μm) containing the proteinosome population loaded with 150 kDa FITC‐dextran was irradiated for 5.5 s using the 405 nm laser on the confocal microscope. The population was then imaged over the next *ca*. 8 min and the fluorescence intensity signal from both RITC‐tagged BSA/PNIPAAm nanoconjugate (red fluorescence) and FITC‐tagged dextran (green fluorescence) was monitored. As can be seen in Figure 3(d), at *t*=0 s, the green fluorescent dextran was contained entirely within the proteinosome membrane, whereas the red fluorescence channel showed well formed proteinosome membranes with an excess of RITC‐tagged BSA/PNIPAAm nanoconjugate trapped and crosslinked within them. During the irradiation time, a rapid decrease of the red fluorescence intensity was observed and was associated with the diffusion and dilution of BSA/PNIPAAm nanoconjugates into the bulk aqueous solution (Figure 3g). After UV irradiation, the red fluorescence intensity remained constant at a value *ca*. 50 % of its initial intensity, whereas the area surrounding the proteinosome membranes gave off a green fluorescent signal (that reached a maximum after *ca*. 90 s) which we attributed to the diffusion of 150 kDa FITC‐dextran out of the proteinosomes (Figure 3g, Supporting Information Figure S32). This was caused by the radiation‐induced breakage of some PEG‐*o*‐NB‐NHS crosslinks, with subsequent release of some BSA/PNIPAAm nanoconjugates and concomitant membrane pore formation. After the initial 90 s, the green fluorescent response started to progressively reduce as the FITC‐dextran diffused away from the immediate vicinity of the proteinosomes into the bulk aqueous media. Whilst we acknowledge that photobleaching of the RITC tag of the BSA/PNIPAAm nanoconjugate could occur during these experiments (see Figure 2b and g, grey and red plots, respectively), the effect can be considered as negligible, as shown by the red plots of red fluorescence intensity over time in Figure 3(g–i).

To investigate the mechanism of dextran release as a function of cargo molecular weight, we performed the same experiment, with the exception that we used a proteinosome population loaded with 2,000 kDa FITC‐dextran instead of the proteinosome population loaded with 150 kDa FITC‐dextran (Figure 3e, h; Supporting Information Figure S33). Like in the previous experiment, during irradiation a rapid decrease of the red fluorescence intensity was observed, caused by pore formation and the concomitant diffusion of RITC‐tagged BSA/PNIPAAm nanoconjugates into the bulk aqueous solution. Following UV irradiation, the red fluorescence intensity remained constant at a value *ca*. 50 % of its initial intensity, whereas now no increase in the green fluorescence intensity in the area around the proteinosome membrane (associated with the release of FITC‐dextran) was observed. In order to confirm our hypothesis, we irradiated another area of the same sample for 15.5 s instead of 5.5 s and monitored the fluorescence intensity of RITC and FITC over time (Figure 3f, I; Supporting Information Figure S34). This time, after irradiation, the red fluorescence intensity remained constant at *ca*. 30 % of its initial intensity, indicating a higher loss of RITC‐tagged BSA/PNIPAAm nanoconjugate and, consequently, the formation of larger pores. The green channel instead showed a rapid increase of the green fluorescence intensity in the regions immediately outside the proteinosome membranes. This signal reached a maximum after *ca*. 47 s and then started to decrease due to the diffusion and dilution of the dextran polymer into the bulk aqueous phase. We attributed this observation to the light‐induced formation of larger pores in the proteinosome membrane.

Taken together, these experiments demonstrate that light can be used as an effective tool to control the release of molecular cargo from a proteinosome membrane based on its molecular weight, favouring first the release of low molecular weight molecules, then those with high molecular weight. These data also align well with our theoretical model that describes the porosity of the proteinosome membrane. A 5.5 s irradiation period leads to an increase in membrane permeability sufficient to allow efflux of 150 kDa dextran but not 2,000 kDa; 15.5 s of irradiation also allows efflux of the polymer with the highest molecular weight. The hydrodynamic radii of 150 and 2,000 kDa dextran are 16.5±1.2 nm and 20.1±1.4 nm, respectively (Supporting Information Figure S35). The release of one single nanoconjugate from the membrane (following crosslinker cleavage) would leave a pore of size in the range *d* to (*d+ξ*), i. e., 15.3–25.2 nm, large enough to allow through 150 kDa dextran, but not 2,000 kDa dextran. If longer irradiation led to the release of two adjacent nanoconjugates from the membrane, then the resultant, larger pore would be twice as wide – i. e., >40 nm, allowing efflux of 2,000 kDa dextran, in good agreement with the experimental data in Figure 3(d–i).

## Conclusions

We have synthesised a novel, photocleavable, PEG‐based crosslinker reactive towards biologically relevant nucleophiles and synthesised from commercial starting materials in 4 steps following a simple synthetic route. Our crosslinker is stable for months when stored appropriately and may be used to produce covalently crosslinked proteinosomes, which are stable over a similar time scale. We have shown that our novel photocleavable crosslinker allows for the photopatterning of proteinosome populations and for the controlled formation of membrane pores, allowing the controlled release of macromolecular cargoes based on their molecular weight. We demonstrated the latter concept using dextran, but we expect and envision that this methodology could be expanded to include a wide range of possible macromolecules (e.g., DNA, enzymes, peptides and antibodies). Moreover, our theoretical proteinosome membrane model could provide a basis for the design and synthetic construction of proteinosomes with different permeability properties, using organic synthesis to tune the dimensions of the protein‐polymer nanoconjugate starting material. Furthermore, we believe that it should be possible to generate a more homogeneous response to light stimuli by more precisely controlling the proteinosome size distribution through the use of microfluidics.^[18]^

Given the wide‐ranging usage of proteinosomes in bottom‐up synthetic biology,^[19]^ we expect that our new methodology of selective release of molecular cargoes based on molecular weight could be used to advance the development of networks of communicating protocells. The photocleavable polymer described above could have widespread use, especially considering its excellent reactivity to primary amines – highly prevalent in proteins and other biomacromolecules. We also envision that our novel crosslinker could be used in contexts beyond bottom‐up synthetic biology, in areas such as functional hydrogels, smart polymers and materials, soft materials chemistry and drug delivery.

## Conflict of interest

The authors declare no conflict of interest.

1

## Supporting information

As a service to our authors and readers, this journal provides supporting information supplied by the authors. Such materials are peer reviewed and may be re‐organized for online delivery, but are not copy‐edited or typeset. Technical support issues arising from supporting information (other than missing files) should be addressed to the authors.

Supporting Information

## Data Availability

The data that support the findings of this study are available from the corresponding author upon reasonable request.
