# Supplementary material for: A Photo‐degradable Crosslinker for the Development of Light‐responsive Protocell Membranes
Source: Chemistry. 2023 Sep 22;29(61):e202302058. doi: 10.1002/chem.202302058 (PMC10946628; doi:10.1002/chem.202302058)
Supplement: Supplementary file 1 — Supporting Information [file CHEM-29-0-s001.pdf]

Chemistry–A European Journal

Supporting Information

A Photo-degradable Crosslinker for the Development of Light-responsive Protocell Membranes

Patrick J. Grimes, Mary Jenkinson-Finch, Henry E. Symons, Wuge H. Briscoe, Sebastien Rochat, Stephen Mann,* and Pierangelo Gobbo*

Contents

1. Supplementary methods 2
1.1. Materials and methods 2
1.2. Synthesis of Photocleavable Crosslinker 4
1.2.1 *o*-NB-NHS **(1)** 4
1.2.2. $(PEG)_{31}$-bis(*o*-nitro benzyl) **(2)** 4
1.2.3. $(PEG)_{31}$-bis([*o*-nitro benzyl] N-succinic acid) **(3)** 5
1.2.4. $(PEG)_{31}$-bis([*o*-nitro benzyl] N-succinimidyl succinate) **(4)** (PEG-o-NB-NHS crosslinker) 5
1.3. Conjugation of FITC/RITC to BSA 6
1.4. BSA cationisation 6
1.5. Conjugation of PNIPAAm to cationised BSA 7
1.6. Synthesis of *o*-NB proteinosomes 7
1.7. *o*-NB proteinosome photolysis 8
1.8. Measurement of laser power-dependent rate of *o*-NB proteinosome photolysis 8
1.9. Measurement of laser scan speed-dependent rate of proteinosome photolysis 9
1.10. Monitoring of photo-induced degradation of an *o*-NB proteinosome population by FACS 9
1.11. Photo-generation of proteinosome patterns 9
1.12. Synthesis of *o*-NB proteinosomes for MWCO determination 10
1.13. Selective cargo release from *o*-NB proteinosomes 10
2. Mathematical modelling of the proteinosome membrane to find mesh size 12
3. Supplementary Figures 18
4. Supplementary Videos 38
5. Supplementary References 38

1. Supplementary methods

1.1. Materials and methods

The following reagents were purchased and used as received. Poly(ethylene glycol)bis(3-aminopropyl) (PEG-diNH$_2$, M$_n$ 1500) , N,N'-dicyclohexylcarbodiimide (DCC) (99%),4-[4-(1-Hydroxyethyl)-2-methoxy-5-nitrophenoxy]butyric acid, 4-dimethylaminopyridine (DMAP) (>99%), N,N-diisopropylethylamine (DIPEA) (>99%), triethylamine (TEA) (>99%), deuterated methanol (CD_3OD) (99.8 atom %), dimethylformamide (DMF), deuterated chloroform ($CDCl_3$) (99.8 atom %), bovine serum albumin (BSA), fluorescein isothiocyanate (FITC), Rhodamine-B isothiocyanate (RITC), fluorescein isothiocyanate-dextran (FITC-dextran) (MW 40, 70, 150 and 2,000 kDa), 1,6-diaminohexane, and PEG-[bis(N-succinimidyl succinate)] (PEG-diNHS) were purchased from Sigma-Aldrich. Succinic anhydride was purchased from Merck. N-hydroxysuccinimide (NHS) (98+%) and 1-ethyl-3-(3-dimethylaminopropyl)carbodiimide (EDAC) (98+%) were purchased from Alfa Aesar. Dialysis tubing with MWCO 1,000 or 12,000–14,000 Da was purchased from Millipore. All solvents were purchased from commercially available sources and used without further purification. Water was filtered using a MilliQ integral 3 system before use. PNIPAAm (M_n = 8,745, M_w = 9,620 Da, $Đ_M$ = 1.1) was synthesised according to literature procedures.[1]

^{1}H and ^{13}C NMR spectra were obtained using Varian 400 MHz or 500 MHz spectrometers, indicated below each NMR spectra. ^{1}H NMR spectra are reported as δ in units of parts per million (ppm) relative to chloroform (δ 7.26, s) or methanol (δ 4.87, s). Multiplicities are reported as follows: s (singlet), d (doublet), t (triplet), q (quartet), p (quintuplet) and m (multiplet). Coupling constants are reported as *J* values in units of Hertz (Hz). The number of protons (n) is reported as nH, and based on spectral integration values. ^{13}C NMR are reported as d in units of parts per million (ppm) relative to $CDCl_3$ (δ 77.16, t) or CD_3OD (δ 49.00, septet). All of the spectra were processed using Mestrenova.

MALDI spectra were recorded on a Bruker UltrafleXtreme (TOF-TOF). Alpha-cyano-4-hydroxycinnamic acid (7 mg mL^{-1} in CH_3CN/0.1% v/v TFA) was used as the matrix solution for polymer samples. The matrix solution was mixed with the polymer sample (1-2 mg mL^{-1} in CH_3CN/0.1% v/v TFA) in 1:1 v/v ratio. For proteins and protein-polymer nanoconjugate samples, the matrix solution was made using 3 equivalents dihydroxyacetophenone in EtOH (20.3 mg mL^{-1}) and 1 equivalent of diammonium hydrogen citrate in water (18 mg mL^{-1}). To prepare the sample, a 1:1:1 v/v mixture of protein in water (1 mg mL^{-1}), 2 % trifluoracetic acid solution in water, and the matrix solution was used.

Gel permeation chromatography was carried out using a Viscotek VE 2001 Triple-Detector Gel Permeation Chromatograph equipped with an automatic sampler, a pump, an injector, an

inline degasser, and a 30 °C column oven. The elution columns consisted of styrene/divinylbenzene gels with pore sizes between 500 Å and 100,000 Å. Detection was conducted with a VE 3580 refractometer, a four-capillary differential viscometer, and 90° and low angle (7°) laser light (λ0= 670 nm) scattering detectors, VE 3210 & VE 270. HPLC grade THF (Fisher)/2.8 mM. Tertbutylammonium bromide was used as the eluent, with a flow rate of 1.0 mL min^{-1}. The calibration was conducted using polystyrene standards form Sigma-Aldrich. Samples were prepared by dissolving the polymer in THF at a concentration of 2 mg mL^{-1} and were filtered through a PTFE membrane with a 0.45 μm pore size before analysis.

Infrared spectra were recorded on a PerkinElmer Spectrum One FT-IR spectrometer. The backgrounds were automatically subtracted from each spectrum.

UV-Vis spectra were recorded using a PerkinElmer Lambda 35 spectrometer and 1 cm quartz cuvettes. For polymers, solutions of 0.1 mM were prepared using phosphate buffer solution (PBS) (0.1 M, pH 7.4). For proteins and protein/polymer nanoconjugates, 1 mg mL^{-1} solutions were prepared in water. The background was automatically subtracted from each spectrum.

Zeta potentials and number-weighted size distributions were measured using a ZETASIZER Nano series instrument (Malvern instruments, UK) using solutions of 0.1 mg mL^{-1} in water.

Fluorescent microscopy images were obtained on a Leica DMI3000 B manual inverted fluorescence microscope at x10, x20, and x63 magnification.

Confocal laser scanning microscopy (CLSM) images were obtained on either a Leica SP5-II AOBS confocal laser scanning microscope with “adaptive focus control” to correct focus drift during time courses, or an SP5 AOBS confocal laser scanning microscope attached to a Leica DM I6000 inverted epifluorescence microscope, at magnifications of x20 (HC PL APO CORR oil lens, working distance 0.17-0.26 mm, numerical aperture 0.7) or x40 (PL APO CS oil lens, 0.22 mm working distance, numerical aperture 1.3). The scan rate is 400 Hz for all experiments. Typically, 20 μL of a dispersed proteinosome sample was taken and imaged on a confocal slide, or in a sealed channel slide. For CLSM images, FITC and RITC labelled proteinosomes were imaged using a 65 mW argon laser (488 nm) or a 20 mW solid state yellow laser (561 nm). Fluorophores were excited by using specific filters with the following excitation (λ_{ex}) and emission wavelength cut offs (λ_{em}); FITC, λ_{ex} = 488 nm, cut off 520 nm; RITC, λ_{ex} = 561 nm, cut off 590 nm. Image analysis was performed with FIJI.

1.2. Synthesis of Photocleavable Crosslinker

1*.2.1* o-*NB-NHS* ***(1)***

4-[4-(1- Hydroxyethyl)-2-methoxy-5-nitrophenoxy]butyric acid (200 mg, 669 µmol, 1.0 eq.) was dissolved in dry DMF under an atmosphere of nitrogen and cooled to 0 °C. EDAC (78 mg, 502 µmol, 1.5 eq.) and *N*-hydroxysuccinimide (58 mg, 502 µmol, 1.5 eq.) were added to the solution before it was degassed and left to stir overnight in darkness (0 °C to r.t.). The solvent was removed under reduced pressure to give a yellow oil which was dried under high vacuum. The dried oil was redissolved in ethyl acetate (30 mL) and washed with deionised water (6 x 10 mL). The combined organic fractions were dried over anhydrous $MgSO_4$ which was removed *via* gravity filtration. The solvent was removed under reduced pressure and the resulting yellow oil was dried under high vacuum. The product was obtained as a yellow solid (233 mg, 583 µmol, 88%).

^{1}H NMR (400 MHz, CD_2Cl_2) δ 7.57 (s, 1H, **e**), 7.31 (s, 1H, **f**), 5.52 (q, 1H, **h**), 4.13 (t, 2H, **d**), 3.96 (s, 3H, **g**), 2.87 (t, 2H, **b**), 2.81 (s, 4H, **a**), 2.29-2.22 (m, 2H, **c**), 1.52 (d, 3H, **i**). ^{13}C NMR (500 MHz, $(CD_3)_2SO$) δ 170.23 (C2), 168.67 (C3), 153.51 (C14), 146.00 (C7), 138.85 (C9), 138.31 (C10), 109.19 (C8), 108.65 (C13), 67.19 (C6), 63.91 (C11), 56.10 (C15), 26.91 (C4), 25.45 (C1), 25.15 (C12), 23.93 (C5). IR ν_{max} (cm^{-1}) 2958 (alkane C-H), 1460 (aromatic N-O stretch), 1361 (aromatic N-O stretch), 1156 (C-O stretch).

*1.2.2. (PEG)$_{31}$-bis(*o*-nitro benzyl)* ***(2)***

PEG-bis(-amine) (196 mg, 98 µmol, 1.0 eq., 50 mgmL^{-1} in dry DMF over 3 Å molecular sieves) was decanted and dry triethylamine (15 mg, 147 µmol, 1.5 eq.) was added under an atmosphere of nitrogen. The solution was degassed before a separate solution of *o*-NB-NHS **(1)** (116 mg, 293 µmol, 3.0 eq.) in dry DMF was added. The reaction mixture was stirred overnight at r.t. and in darkness. The product solution was added to rapidly stirring diethyl ether (200 mL) and a cream precipitate formed on addition. The suspension was stirred at 0 °C for 20 min before being centrifuged at 3260 g for 10 min. The clear supernatant was discarded and the remaining cream/yellow pellets were dried under high vacuum. The pellets were dissolved in MilliQ water (10 mL) and the resultant solution was dialysed (using dialysis tubing with MCWO = 1 kDa) against MilliQ water (3 x 3L) for 4 h. The product solution was lyophilised to afford a yellow solid (177 mg, 66 µmol, 67%).

^{1}H NMR (400 MHz, Methanol-*d*$_4$) δ_H 7.60 (d, *J* = 3.0 Hz, 2H, ArH), 7.40 (d, *J* = 8.3 Hz, 2H, ArH), 5.46 (q, *J* = 6.4 Hz, 2H), 4.09 (t, *J* = 6.2 Hz, 4H), 3.97 (s, 6H), 3.65 (m), 3.52 (t, *J* = 6.1 Hz, 4H) 3.29 (t, *J* = 6.8 Hz, 4H), 2.39 (t, *J* = 7.4 Hz, 4H), 2.11 (p, *J* = 7.1 Hz, 4H), 1.77 (m, 4H), 1.48 (d, *J* = 6.2 Hz, 6H). ^{13}C NMR (500 MHz, Methanol-*d*$_4$) δ_C 175.2, 163.9, 144.4, 148.3, 140.7, 139.0, 110.0, 73.7, 69.9, 66.1, 56.8, 37.9, 33.5, 30.3, 26.5, 25.3. IR ν_{max} (cm^{-1}) 2882.6 (Alkane C-H stretch), 1516.7 (Aromatic N-O stretch), 1101.6 (C-O stretch). MALDI-TOF (m/z) found: M_n 2023, estimated PDI 1.01.

1.2.3. (PEG)$_{31}$-bis([o-nitro benzyl] N-succinic acid) ***(3)***

Compound **(2)** (177 mg, 69 µmol, 1.0 eq.), succinic anhydride (138 mg, 1380 mmol, 20.0 eq.) and DMAP (8 mg, 69 µmol, 0.4 eq.) were dissolved in dry DMF (*ca*. 5 mL) under an atmosphere of N_2. The solution was heated to 50 °C and stirred in the dark for 18 h. The resulting amber solution was added dropwise to ice-cold diethyl ether (*ca.* 250 mL) to precipitate the polymer. The mixture was stirred in the dark and at 0 °C for 2 h before being transferred to falcon tubes. The mixture was then centrifuged for 10 min at 3260 g to isolate the solid polymer. The colourless, transparent supernatant was discarded and the remaining pellets were dried under a flow of N_2. The dried pellets were then dissolved in MilliQ water and the solution was transferred to dialysis tubing (MWCO = 1 kDa). The dissolved impure product was then dialysed against MilliQ water (*ca*. 3 L) for 4 h. The MilliQ water was replaced three times during dialysis. The product solution was removed from the dialysis tubing and was transferred to pre-weighed flacon tubes and lyophilised. **(3)** was isolated as a cream solid (164 mg, 57 µmol, 82%).

^{1}H NMR (400 MHz, Methanol-*d*$_4$) δ_H 7.62 (s, 2H, ArH), 7.17 (s, 2H, ArH), 6.37 (q, *J* = 6.5 Hz, 2H), 4.11 (t, *J* = 6.1 Hz, 4H), 4.01 (s, 6H), 3.64 (m, 344H), 3.53 (m, 4H), 3.27 (m, 4H), 2.65 (m, 4H), 2.47 (t, *J* = 7.0 Hz, 4H), 2.41 (t, *J* = 7.4 Hz, 4H), 2.12 (p, *J* = 6.9 Hz, 4H), 1.77 (m), 1.62 (d, *J* = 6.4 Hz, 6H). ^{13}C NMR (500 MHz, Methanol-*d*$_4$) δ_C 76.0, 175.2, 174.5, 173.3, 155.6, 148.7, 141.0, 134.3, 110.0, 109.6, 71.5, 69.9, 69.8, 57.0, 37.9, 33.5, 31.8, 30.6, 30.3, 26.4, 22.2. IR ν_{max} (cm^{-1}) 2882.7 (Alkane C-H stretch), 1732.6 (Carbonyl C=O stretch), 1520.5 (Aromatic N-O stretch), 1103.1 (C-O stretch). MALDI-TOF (m/z) found: M_n = 2164, estimated PDI 1.01.

1.2.4. (PEG)$_{31}$-bis([o-nitro benzyl] N-succinimidyl succinate) ***(4)*** *(PEG-o-NB-NHS crosslinker)*

PEG-bis-photocleavable group-diacid (164 mg, 60 μmol, 1.0 eq.), dicyclohexylcarbodiimide (35 mg, 180 μmol, 3.0 eq.) and N-hydroxysuccinimide (21 mg, 180 μmol, 3.0 eq.) were placed under an atmosphere of N_2 and were dissolved in dry dichloromethane (*ca*. 5 mL). The resulting solution was stirred at room temperature, in the dark and under nitrogen overnight. A white precipitate formed in the reaction vessel which was removed *via* filtration. The resulting amber solution was added to ice-cold diethyl ether in falcon tubes and a cream precipitate formed on addition. The mixture was centrifuged at 3260 g for 10 minutes. Brown pellets were formed with a clear, colourless supernatant. The ether supernatant was discarded and the remaining solid product was dried under a flow of N_2. The dried solid was then dissolved in a minimum volume of dichloromethane and was transferred to a pre-weighed vial and dried under high vacuum. The final product was afforded as a waxy brown solid (165 mg, 57 μmol, 95%).

^{1}H NMR (500 MHz, Methanol-*d₄*) δ_H 7.61 (d, *J* = 4.6, 2H, ArH), 7.17 (d, *J* = 4.3, 2H, ArH), 6.37 (q, *J* = 6.0, 5.3 Hz, 2H), 4.2-4.11 (m, 4H), 3.99 (m, 6H), 3.65 (m, 306H), 3.26 (m, 4H), 2.88 (s, 8H), 2.59 (m, 4H), 2.47 (m, 4H), 2.42 (m, 4H), 2.13 (m, 4H), 1.77 (m, 4H), 1.64 (m, 6H).

^{13}C NMR (500 MHz, Methanol-*d₄*) δ_C 180.0, 176.1, 175.1, 174.5, 173.0, 155.6, 148.7, 141.1, 134.1, 110.1, 109.6, 69.9, 69.8, 57.1, 37.9, 33.5, 31.4, 30.4, 30.2, 29.5, 26.4, 22.2. MALDI-TOF (m/z) found: 2175, estimated PDI 1.01.

1.3. Conjugation of FITC/RITC to BSA

BSA (20 mg) was dissolved in 7.4 mL carbonate buffer (pH 8.5, 100 mM) to obtain a 2.7 mg mL^{-1} solution. An aliquot (193.5 μL) of a solution of FITC/RITC in DMSO (1 mg mL^{-1}) was added to the BSA solution and the mixture stirred overnight at 4 °C. The next day the BSA/fluorophore conjugate was dialysed against water for 8 hours, changing the water at least 3 times, and then centrifuged to remove aggregates. The supernatant was taken and lyophilised overnight to give the fluorescently tagged-BSA. Zeta potential: -15.2 mV. MALDI-TOF (m/z) found: 66200. Degree of labelling: 20%.

1.4. BSA cationisation

In a vial, bovine serum albumin (BSA) (18 mg) was dissolved in 1.8 mL of water to make a 10 mg mL^{-1} solution. Separately, a solution of 1,6-diaminohexane (180 mg) was dissolved in water (1.8 mL, 100 mg mL^{-1} solution). The pH of the solution was adjusted to 6-6.5 using NaOH/HCl (5 M). The solution of 1,6-diaminohexane was slowly added to the solution of BSA. The pH was readjusted to 6-6.5. EDAC (9 mg) was dissolved in water (500 μL) and added to the solution of protein. The pH was adjusted to 6-6.5. After 1 hour, in an Eppendorf was weighed a further aliquot of EDAC (9 mg) which was dissolved in water (500 μL) and added to the vial. The solution was left to stir overnight. The next day the reaction mixtures were dialysed and centrifuged (5000 rpm, 5 min) to remove precipitate (crosslinked protein). The supernatant was taken and lyophilised overnight to give the cationised BSA. Zeta potential: +41.5 mV. MALDI-TOF (m/z) found: 69692. Degree of cationisation: 28.8 %.

1.5. Conjugation of PNIPAAm to cationised BSA

In a vial, FITC- or RITC-labelled cationised BSA (20 mg) was dissolved in 10 mL of water to make a 2 mg mL^{-1} solution. In a separate vial, PNIPAAm (20 mg) was dissolved in 10 mL water (2 mg/mL solution) and subsequently added dropwise to the solution of FITC- or RITC-labelled cationised BSA and left to stir at room temperature overnight. The reaction mixture was centrifuged in 50 kDa centrifugal filters and washed 4 times with water to remove unreacted polymer. Finally, the samples were lyophilised to give the FITC- or RITC-labelled BSA/PNIPAAm nanoconjugates. Zeta potential: -7.2×10^{-2} mV. MALDI-TOF (m/z) found: 69692 Da.

1.6. Synthesis of *o*-NB proteinosomes

In a small Eppendorf tube 3 mg *o*-NB crosslinker were dissolved in 30 μL of Na_2CO_3 buffer (pH 8.5, 100 mM) to obtain a 100 mg/mL solution. In a vial, 30 μL of the FITC- or RITC-labelled BSA/PNIPAAm nanoconjugate solution (8 mg mL^{-1} in water) was mixed with 30 μL of the crosslinker solution and swirled gently to obtain a homogenous solution. 1000 μL of oil (2-ethyl-1-hexanol) (0.06 v/v %) was added to this mixture. The vial was vigorously shaken 30 seconds by hand. The solution became turbid. After 60 h storage in darkness, the photo-sensitive proteinosomes sedimented and the upper oil layer was removed from the vial. Subsequently, 1 mL of ethanol/water solution (70 % EtOH/H_2O) was used to dissolve the photo-sensitive proteinosomes before they were dialysed against (70 % EtOH/H_2O) for 4

hours. The dialysis solution was then switched to 35 % EtOH/H_2O and the photo-sensitive proteinosomes dialysed for a further 3 hours. Finally, photo-sensitive proteinosomes were dialysed against water overnight and in darkness. In order to wash broken proteinosomes from the sample, it was left to sediment overnight and the upper layer of water was removed and replaced. This washing procedure was repeated twice more to yield a photo-sensitive proteinosome sample in a final volume of 1 mL.

For the control experiments we adopted the same procedure described above, but instead of using the *o*-NB crosslinker, we used 30 μL of a solution of PEG-diNHS (50 mg mL^{-1}).

1.7. *o*-NB proteinosome photolysis

Typically, in a dark room, 20 μL of a washed sample of RITC-labelled *o*-NB proteinosomes was pipetted into a channel slide before being sealed with silica based vacuum grease to avoid evaporation. The proteinosomes were left to settle for 10 minutes. On a CLSM using the 40x HCX PL APO oil lens, a region of interest was chosen varying from 388 x 388 μm^2 (large population of proteinosomes) to 100 μm^2 (single proteinosome). In a typical experiment, samples were irradiated with a 50 mW diode laser (405 nm, 100 % laser power, 0.648 seconds/frame) for 300 seconds. Frame by frame analysis on the software FIJI software was used to obtain a video. Fluorescence intensity of RITC-labelled *o*-NB proteinosomes was plotted against time to give a graphical representation.

1.8. Measurement of laser power-dependent rate of *o*-NB proteinosome photolysis

Typically, 20 μL of a washed sample of *o*-NB proteinosomes was sealed in a channel slide. On a CLSM using the 40x HCX PL APO oil lens, 561 nm (for RITC-tagged *o*-NB proteinosomes) laser was set to 15 %. A region of interest of 388x388 μm^2 was drawn using the region of interest tool. The 405 nm laser (50 mW) intensity was set between 5 and 100 % (17-2266 μW), pre-bleach was left at 1 iteration, bleach was set to 2000, and post-bleach was set to 1. Images were acquired at 0.648 sec/frame. For data analysis, please see supplementary section 1.9. This was repeated for each of the laser powers.

1.9. Measurement of laser scan speed-dependent rate of proteinosome photolysis

Typically, 20 μL of a washed sample of *o*-NB proteinosomes was sealed in a channel slide. On a CLSM using the 40x HCX PL APO oil lens, the 561 nm (for RITC-tagged *o*-NB proteinosomes) laser was set to 15 %. A region of interest between 388x388 and 100x100 μm^2 was drawn using the region of interest tool. The 405 nm laser (50 mW) intensity was set to 100 %, pre-bleach was left at 1 iteration, bleach was set to 500, and post-bleach was set to 1. Images were acquired by varying the laser scan speed between 0.6 – 0.63 μm s^{-1}.

Fluorescence intensity of 1/10th of the population was measured over time using ImageJ software to obtain fluorescence decay curves over time. The proteinosome degradation time was defined as the intercept point between the linear regressions of the initial decay and the plateau region.

1.10. Monitoring of photo-induced degradation of an *o*-NB proteinosome population by FACS

Typically, 100 μL of a washed FITC-tagged proteinosome sample was transferred into a 1.5 mL Eppendorf tube and irradiated for 1, 15, 30 minutes using a Xenon light source (11.5 mW) with a 365 nm filter, and fitted with a solution probe clamped 0.5 cm above the solution. The solution was diluted 10 times using water. Flow cytometry was performed on a Novacyte 3000 equipped with a 488 nm laser. Thresholds used were as follows: Forward scatter (FSC-H) > 500; side scatter (SSC-H) and fluorescence (FITC-H) ungated. Flow rate: medium, 35 μL min^{-1}. Stop condition: 50 μL.

1.11. Photo-generation of proteinosome patterns

Typically, 20 μL of a washed sample of RITC-labelled *o*-NB proteinosomes was sealed in a channel slide. On a confocal microscope using the 40x HCX PL APO oil lens, the desired pattern was drawn using the region of interest tool. This area was irradiated with a 50 mW diode laser (405 nm, 0.648 seconds/frame). For positive images, the areas around the desired pattern were selectively irradiated, and for negative patterns, the proteinosomes inside the desired area were selectively irradiated.

1.12. Synthesis of *o*-NB proteinosomes for MWCO determination

In order to encapsulate FITC-dextran, 10 μL of a solution of FITC-dextran (40, 70, 150 or 2,000 kDa, 6 mg mL^{-1}, H_2O) was mixed with 30 μL of RITC-labelled BSA/PNIPAAm nanoconjugate solution (8 mg mL^{-1} in water). The *o*-NB crosslinker (2 mg) was dissolved in 20 μL of Na_2CO_3 buffer (pH 8.5, 100 mM) and added to the nanoconjugate/dextran mixture to give a final FITC-dextran concentration of 1 mg mL^{-1}. An oil layer of 1-ethyl-2-hexanol (1 mL) was carefully added on top and the vial was shaken vigorously for 30 seconds. The resulting emulsion was left to sediment and crosslink for 48 hours in darkness before being transferred to water as described in supplementary section 1.6. After transfer, the proteinosome sample was washed 3 times as described in supplementary section 1.6 to make sure broken proteinosomes and FITC-dextran in the bulk solution were completely removed.

1.13. Selective cargo release from *o*-NB proteinosomes

In order to encapsulate FITC-dextran, 10 μL of a solution of FITC-dextran (150 or 2,000 kDa, 6 mg mL^{-1}, H_2O) was mixed with 30 μL of FITC- or RITC-labelled BSA/PNIPAAm nanoconjugate solution (8 mg mL^{-1} in water). The *o*-NB crosslinker (2 mg) was dissolved in 20 μL of Na_2CO_3 buffer (pH 8.5, 100 mM) and added to the nanoconjugate/dextran mixture to give a final FITC-dextran concentration of 1 mg mL^{-1}. An oil layer of 1-ethyl-2-hexanol (1 mL) was carefully added on top and the vial was shaken vigorously for 30 seconds. The resulting emulsion was left to sediment and crosslink for 48 hours in darkness before being transferred to water as described in supplementary section 1.6. After transfer, the proteinosome sample was washed 3 times as described in supplementary section 1.6 to make sure broken proteinosomes and FITC-dextran in the bulk solution were completely removed.

Generally, 20 uL of proteinosomes were taken from the bottom of the vial, sealed in a channel slide, and left to sediment to the bottom for 10 minutes. On a CLSM using the 40x HCX PL APO oil lens, the 488 and 561 nm lasers were set to 15 % and optimised to eliminate crossover of the lasers. A 200x200 μm^2 area was drawn in the middle of a 388x388 μm^2 total frame size using the region of interest tool. The 405 nm laser intensity was set to 100 % and 'Fly Mode' was selected. Pre-bleach was left at 1 iteration, bleach was set to 5 (150 kDa) or 12 (2,000 kDa), and post-bleach was set to 200.

Fluorescence intensity of 1/10th of the population was measured over time using ImageJ software to obtain fluorescence decay curves over time. The proteinosome degradation time was defined as the intercept point between the linear regressions of the initial decay and the plateau region.

FITC-dextran release was monitored by measuring the fluorescence intensity of the background immediately outside individual proteinosomes using ImageJ. Once fluorescence intensity curves over time were found, they were normalised to peak at 100.

2. Mathematical modelling of the proteinosome membrane to find mesh size

According to the de Gennes theory,[2] the mesh size (ξ) for a semi-dilute solution of polymers is given by the equation:

$$\xi = a\phi^{-3/4} \qquad \textbf{(Eq. 1)}$$

where $\boldsymbol{a}$ is the monomer length and $\boldsymbol{\phi}$ is the polymer volume fraction – the volume occupied by the polymer molecules as a fraction of the total volume pervaded by the polymers in solution.

However, the proteinosome membrane is more complex than a simple semi-dilute solution of polymer chains; protein-polymer nanoconjugates sit partially submerged within the random web of PEG chains in the membrane, and the membrane itself only occupies a very restricted volume on the surface of the proteinosome. The following describes an attempt to mathematically incorporate these other factors, using the de Gennes equation as a base (Equation 1), to give a more accurate estimation of the mesh size of the proteinosome membrane.

Firstly, $\boldsymbol{a}$ is simply the monomer length of PEG, available from literature sources (0.35 nm).[3] According to de Gennes, the value of $\boldsymbol{\phi}$ can be defined as the ratio of the molecular volume of the PEG crosslinkers (V_L) and the volume pervaded by the crosslinkers in the membrane (solution volume, V_x):

$$\phi = \frac{V_L}{V_x} \qquad \textbf{(Eq. 2)}$$

Based on our proteinosome membrane model (Figure **3a** in main text), the solution volume (V_x) of the crosslinker can be estimated by calculating the volume of the whole aqueous shell of the membrane and removing any volume contribution from the nanoconjugates:

$$V_x = V_s - V_c \qquad \textbf{(Eq. 3)}$$

where V_s is the total volume of the aqueous shell of the proteinosome membrane, and V_c is the combined volume of the nanoconjugates sitting partially submerged in the membrane's aqueous shell. This approximation (Equation 3) assumes that all the crosslinking polymers reside entirely within the aqueous shell of the membrane.

The spherical aqueous shell has a thickness, t, and a radius, R. Making the assumption that the proteinosome is indeed perfectly spherical, and that its membrane thickness is vastly smaller than its radius, the aqueous shell volume (V_s) could be expressed as:

$\boldsymbol{V_s = 4\pi\,(R - t)^2 t}$ or, for $t \ll R$, $\boldsymbol{V_s \approx 4\pi R^2 t}$ **(Eq. 4)**

As nanoconjugates are amphiphilic, and proteinosome synthesis involves the formation of an emulsion with nanoconjugates self-assembled at the oil/water interface, only a portion of each nanoconjugate would be submerged in the aqueous shell of the membrane. The remainder of a nanoconjugate would protrude from the aqueous shell into the oil phase of the emulsion; the extent of this protrusion is determined by the hydrophobic contact angle of the nanoconjugate. As we assumed that nanoconjugates were hard, perfect spheres, this can be expressed as

$\boldsymbol{t = \delta d}$ **(Eq. 5)**

where d is the diameter of the nanoconjugate, δ is the fraction of the nanoconjugate submerged in the aqueous shell of the membrane, and t is the thickness of the aqueous shell. Assuming that the contact angle of the nanoconjugate is equal to 90°, then

$\boldsymbol{t = \frac{1}{2} d}$ **(Eq. 6)**

i.e., $\boldsymbol{\delta = 0.5}$ (if further research provides more precise information on the contact angle, then δ can be adjusted accordingly).

The volume that a single nanoconjugate occupied within the aqueous shell (v_{nc}) could be described by the spherical cap volume of a nanoconjugate. Using the standard equation for the volume of a spherical cap:

$$v_{nc} = \frac{\pi t^2}{3}\left(\frac{3}{2}d - t\right) \qquad \textbf{(Eq. 7)}$$

Using equation (6), this could be simplified further to:

$$v_{nc} = \frac{1}{12}\pi d^3 \qquad \textbf{(Eq. 8)}$$

Each proteinosome membrane is comprised of n nanoconjugates, therefore V_c is expressed as being equal to the submerged volume of a nanoconjugate multiplied by n:

$$V_c = n v_{nc} = \frac{n}{12}\pi d^3 \qquad \textbf{(Eq. 9)}$$

Therefore, the solution volume of the crosslinking PEG polymers, V_x, can be expressed as:

$$V_x = V_s - V_c = 2\pi R^2 d - \frac{n}{12}\pi d^3 = \frac{\pi d(24R^2 - nd^2)}{12} \qquad \textbf{(Eq. 10)}$$

R and d can be determined experimentally; however, the value for n must be estimated, requiring a model of the nanoconjugate within the membrane. We have modelled the nanoconjugates within the membrane as following a hexagonal packing regime. However, we assumed that space would exist between the nanoconjugates as, from experimental evidence, we determined that the proteinosome membrane was permeable to dextran with a hydrodynamic radius greater than the minimum space between the nanoconjugates (if we were to assume perfect hexagonal *dense* packing). Therefore, there must be space, S, between the nanoconjugate spheres filled by the crosslinker in semi-dilute phase. Thus, the hexagonal area pervaded by a nanoconjugate is:

$$a_{nc} = \frac{\sqrt{3}}{2}(d + S)^2 \quad \text{(Eq. 11)}$$

where $(d + S)$ is the hexagonal diameter attributed to each nanoconjugate plus its surrounding space. We then divided our value for the total proteinosome membrane area by the hexagonal area of a single nanoconjugate to get a value for n:

$$n \approx \frac{A}{a_{nc}} = \frac{8\pi R^2}{\sqrt{3}(d+S)^2} \quad \text{(Eq. 12)}$$

R was assessed *via* confocal fluorescence microscopy and was found to be an average of 11.25 μm. $(d+S)$ was determined experimentally, as described in above which gave a range of possible values between 17.6 – 30.2 nm.

Now that the solution volume (V_x) had been estimated, the remaining term needed to calculate ϕ was the molecular volume of the PEG crosslinkers (V_L) (Equation 13). As the crosslinking PEG molecules are in large excess in the initial proteinosome synthesis, we assumed that every reactive site (z) on the nanoconjugate had reacted with a crosslinker. Furthermore, because the crosslinkers are reactive at both ends of the central PEG chain, then the number of crosslinkers per nanoconjugate must equal $\frac{z}{2}$.

However, as the nanoconjugate has been modelled such that $t = \delta d$, then the number of reactive sites (z) must be reduced by a proportion equal to δ. As we have assumed that $\delta = \frac{1}{2}$, then the number of crosslinkers per nanoconjugate must equal $\frac{z}{4}$. We multiplied this by n (the total number of nanoconjugates per proteinosome) to get a value for the total number of the crosslinker PEG chains.

We defined the absolute mass per crosslinker as m and its density as ρ and could then estimate the molecular volume of a single crosslinking polymer as:

$$V_L = \frac{m}{\rho} = \frac{M}{\rho N_A} \quad \text{(Eq. 13)}$$

where M is the molecular weight and N_A is Avogadro's number. We assumed here that the crosslinker density was equivalent to that of 2 kDa polyethylene glycol, which comprises the majority of the mass of the compound.

Finally, ϕ could be calculated using the above expressions for V_L and V_x. The term $\frac{nz}{4}$ is used as a scaling factor to get a value for V_L of all the crosslinkers in a single proteinosome membrane. Here, n is the number of nanoconjugates and z is the number of reactive amine groups on the surface of each nanoconjugate.

$$\phi = \frac{\left(\frac{nz}{4}\right)v_L}{V_x} \qquad \textbf{(Eq. 14)}$$

With an expression for volume fraction, and values for n, z, and ρ, it was possible to obtain an estimation for the mesh size of the proteinosome membrane.

Dynamic light scattering (DLS) was used to experimentally determine a range of values for the diameter of a nanoconjugate, d, of 13.6 ± 1.6 nm. Equations (5) and (6) can then be used to give a value for membrane thickness of between 6.0 and 7.6 nm.

Confocal microscopy was used to directly measure the value for R (the radius of the proteinosome), giving an average of 11.25 µm. Combining this with the nanoconjugate diameter in equation (4), we calculated the volume of the membrane aqueous shell, V_s to be between 9.54 x 10^9 and 1.21 x 10^{10} nm^3.

Next we employed equation (8) to calculate v_{nc}, the submerged aqueous volume of an individual nanoconjugate; between 452 and 919 nm^3. Using these values, we could work out the total volume occupied by the nanoconjugates (V_c) within the aqueous shell of the proteinosome membrane by multiplying v_{nc} by n (equation 9).

To find n, we used equation (12) which includes only one unknown term, s. A range of possible values can be attributed to s as a result of the experimental work. We observed the minimum molecular weight of fluorescently tagged dextran that could freely diffuse through the proteinosome membrane was 40 kDa. As we determined 40 kDa dextran had a hydrodynamic radius of 5.6 ± 1.6 nm (*via* DLS, Figure **S35**) then the minimum inter-conjugate distance was 5.6 nm. The maximum inter-conjugate distance was theorised as the contour length of the crosslinking polymer – 15 nm. Therefore, $(d + S)$ can be estimated as between 17.6 and 30.2

nm. Using these values in equation (11), we calculated values for a_{nc} of between 268 and 790 nm^2. This allowed to us obtain a range between 2.01 x 10^6 and 5.93 x 10^6 nanoconjugates per proteinosome.

Using equation (10), we calculated V_x as between 6.3 x 10^9 and 1.02 x 10^{10} nm^3.

The remaining term needed to determine ϕ was V_L. To calculate this value, as discussed above, we employed equation (13), using 1.21 g cm^{-3} as the assumed density (ρ) of the crosslinker to give V_L as 2.74 nm^3. Finally, we obtained a range of values for ϕ, using equation (14). We used our previously calculated values for V_x, and had to estimate z based on the experimental conditions used in protein-polymer nanoconjugate and proteinosome synthesis. the crosslinkers in the proteinosome membrane were present in approx. 7 times excess during proteinosome synthesis. As z is equal to the number of crosslinking sites – *i.e.*, the number of free amine groups on the nanoconjugate surface – this could be estimated by taking into account the chemical structure. The nanoconjugate has 59 amines provided by lysine residues on the BSA protein core, plus the 29 cationised aspartate and glutamate residues, minus three binding sites used up by the conjugation of the PNIPAAm chains. This gives a total number of binding sites for the crosslinkers, z, of 85. As described above, we divided z by 4 to reflect the chemical structure of the proteinosome membrane.

Inputting all of these numbers into equation (14), we obtained values for ϕ of between 0.012 and 0.050. Using literature values for PEG monomer length (a = 0.35 nm) and equation (1), we calculated the mesh size, ξ, of the crosslinkers in the proteinosome membrane to be between 3.29 and 9.98 nm. This aligns remarkably well with our experimental results; the proteinosome membrane was able to effectively encapsulate 70 kDa dextran (hydrodynamic radius = 12.3 nm) but was permeable to 40 kDa dextran (hydrodynamic radius = 5.6 nm).

4. Supplementary Figures

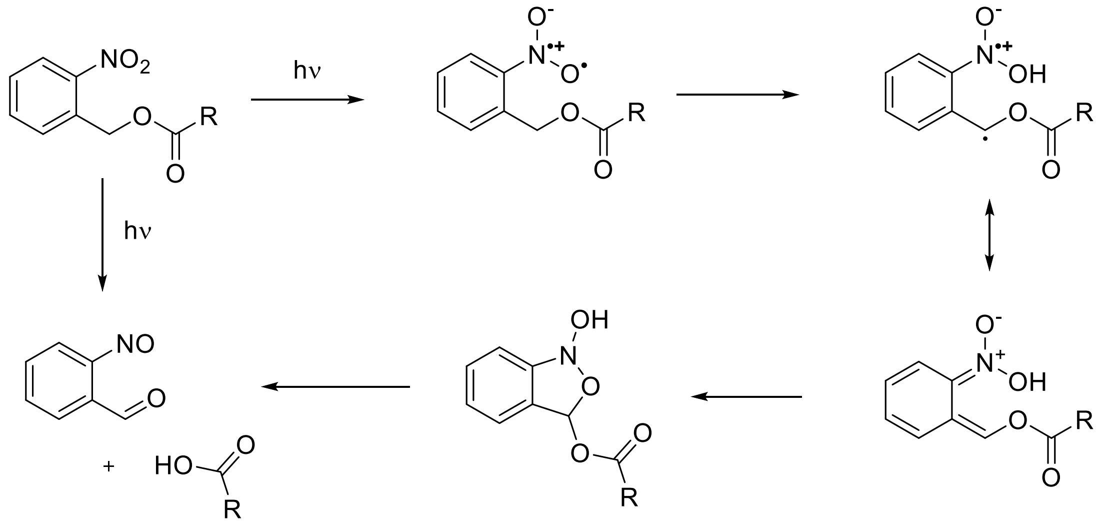

Supplementary Figure S1 Scheme describing the general mechanism of *o*-NB cleavage under UV light.

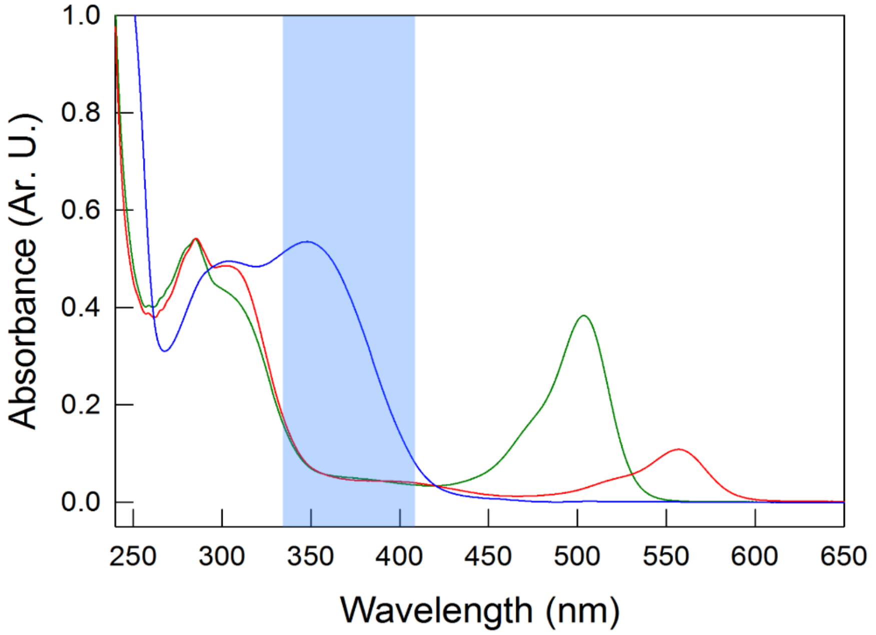

Supplementary Figure S2 UV-Vis spectrum of FITC-tagged nanoconjugate (green line), RITC-tagged nanoconjugate (red line), and of *o*-NB functionalised crosslinker (blue line). Highlighted with a blue band is the ideal wavelength window of irradiation, in which there is minimal absorbance from the FITC- or RITC-labelled BSA/PNIPAm nanoconjugate constituting the proteinosome membrane and maximum absorbance from the *o*-NB moiety.

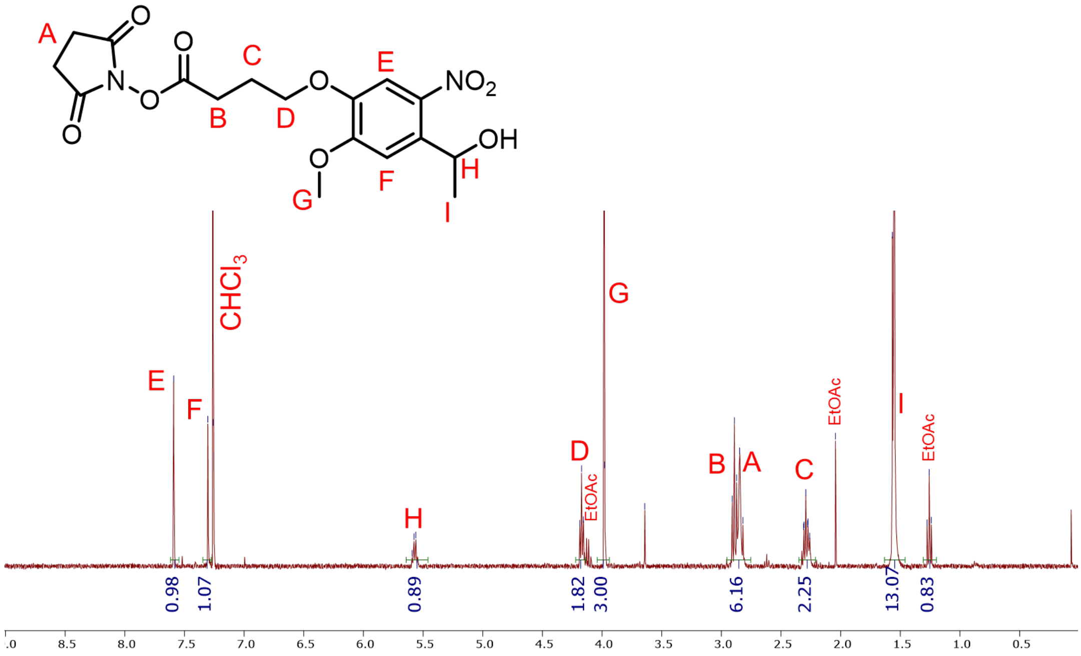

Supplementary Figure S3 ^{1}H NMR spectrum of compound **1** recorded on 400 MHz NMR spectrometer in CD_2Cl_2 and calibrated against residual dichloromethane.

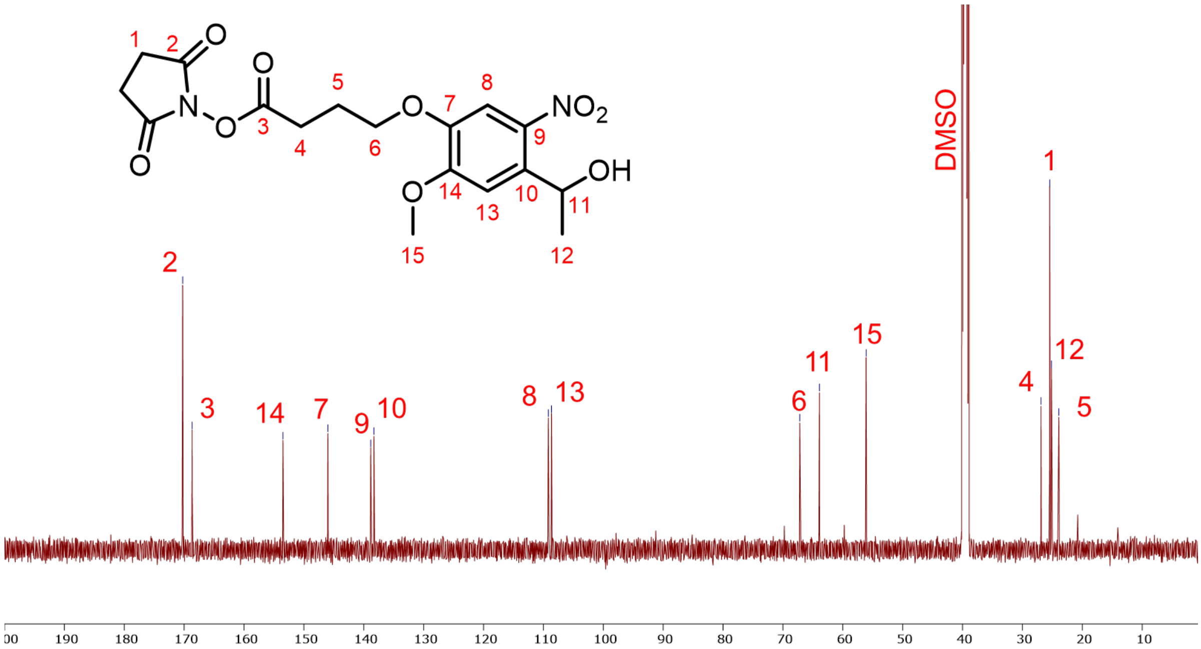

Supplementary Figure S4 ^{13}C NMR spectrum of compound **1** recorded on 500 MHz NMR spectrometer in DMSO-d_6 and calibrated against residual DMSO.

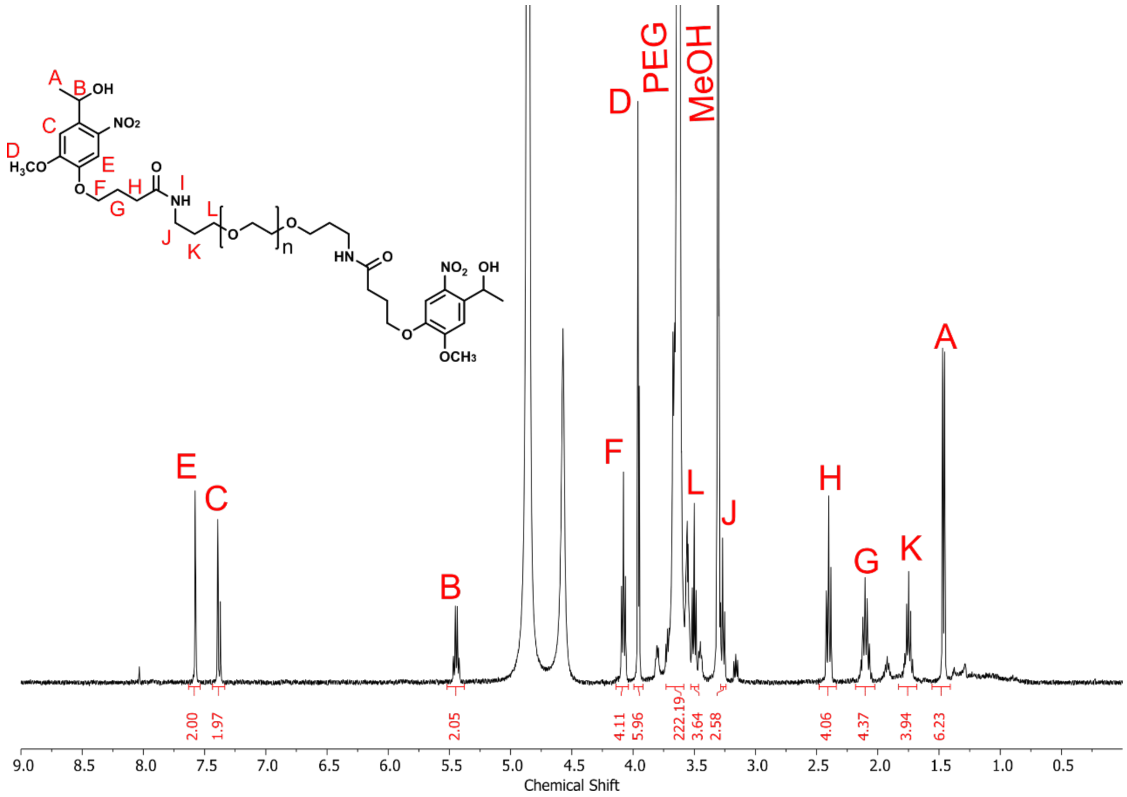

Supplementary Figure S5 ^{1}H NMR spectrum of **(2)** recorded on 400 MHz NMR spectrometer in CD_3OD and calibrated against residual methanol.

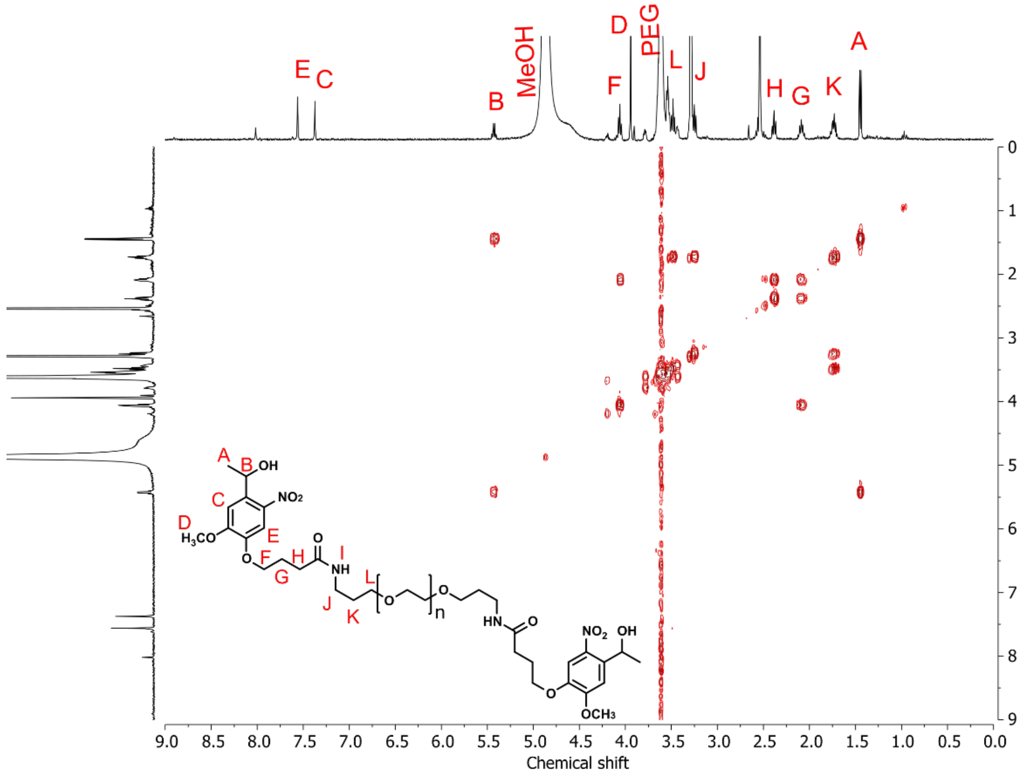

Supplementary Figure S6 2D ^{1}H COSY NMR spectrum of **(2)** recorded on a 400 MHz NMR spectrometer in CD_3OD and calibrated against residual methanol.

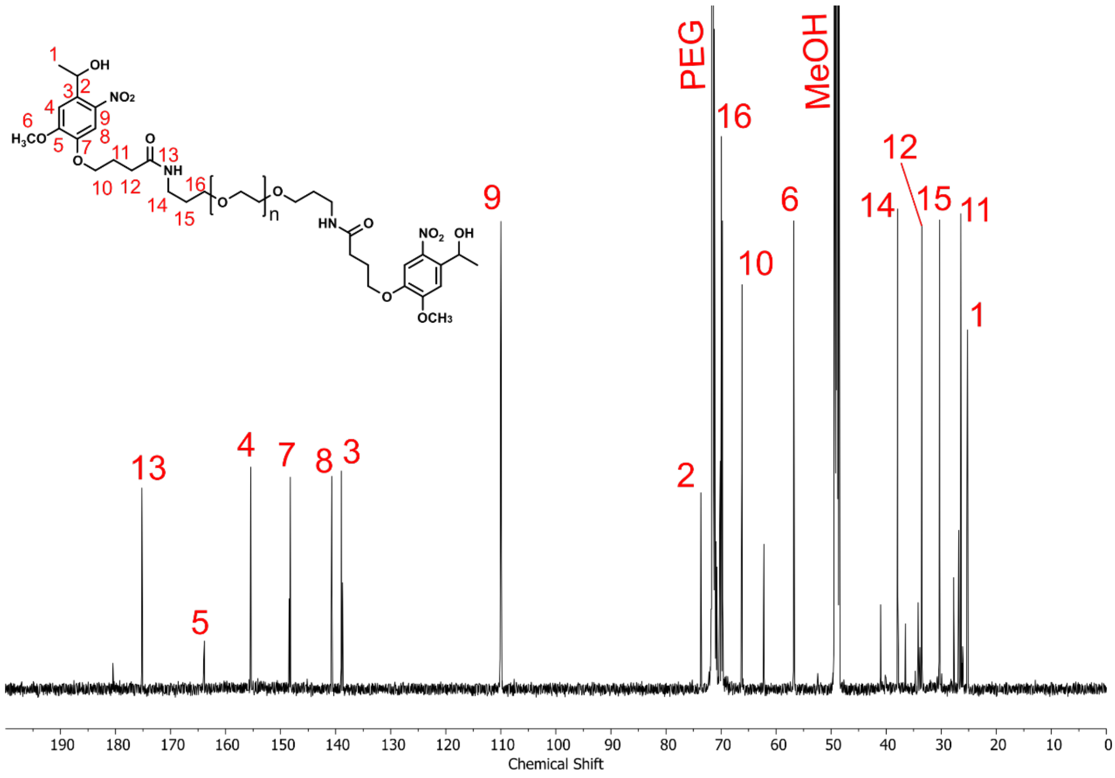

Supplementary Figure S7 ^{13}C NMR spectrum of **(2)** recorded on 500 MHz NMR spectrometer in CD_3OD and calibrated against residual methanol.

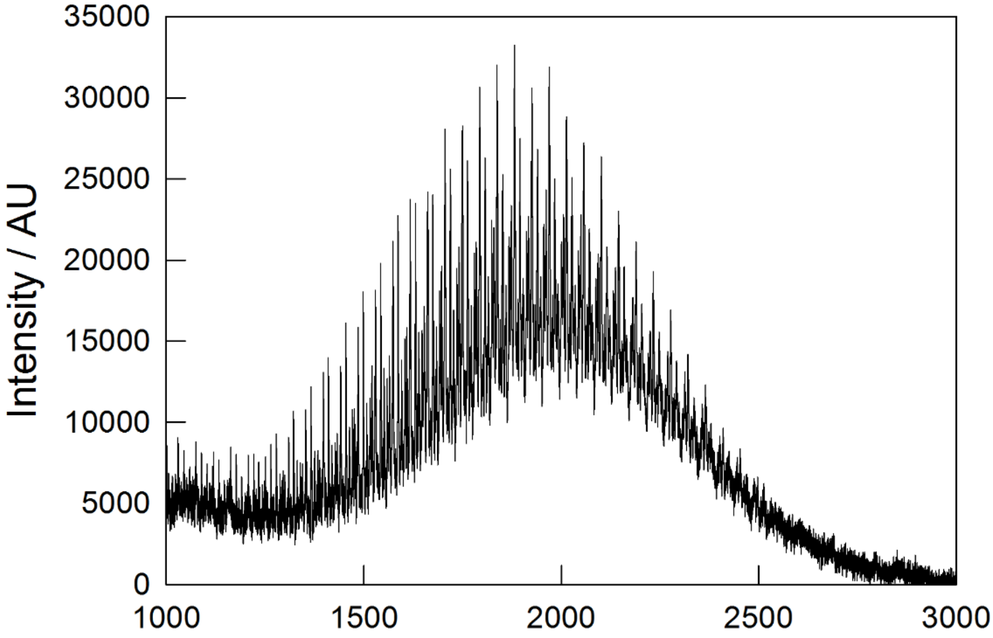

Supplementary Figure S8 MALDI-TOF spectrum of **(2)** recorded using alpha-cyano-4-hydroxycinnamic acid (7 mg/mL in ACN/0.1%TFA) as the matrix solution, on a Bruker UltrafleXtreme (TOF).

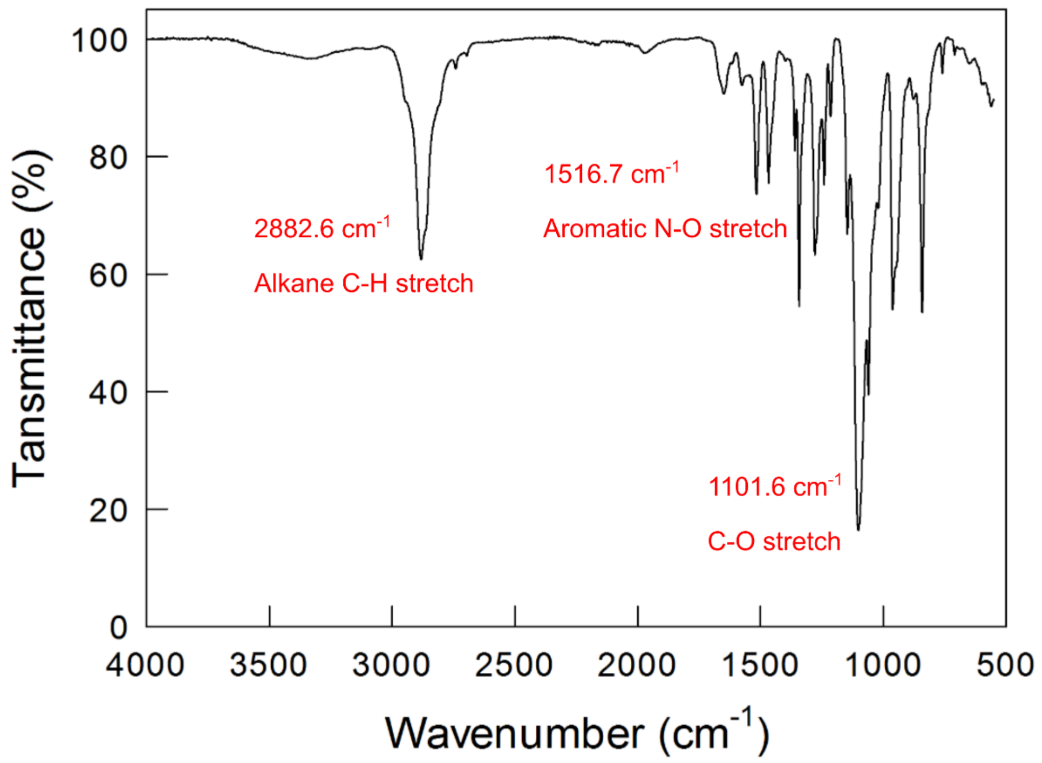

Supplementary Figure S9 FT-IR spectrum of **(2)** recorded on a PerkinElmer Spectrum One FT-IR spectrometer.

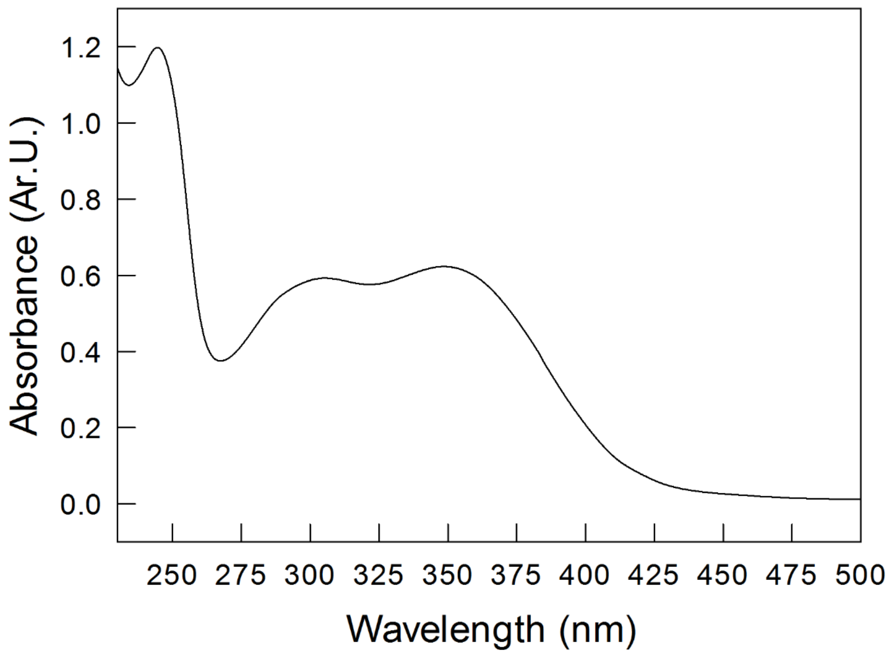

Supplementary Figure S10 UV-Vis spectrum of a 0.1 mM solution of **(2)** in PBS buffer (0.1 M, pH 7.4). Spectrum recorded on a PerkinElmer Lambda 35 spectrometer.

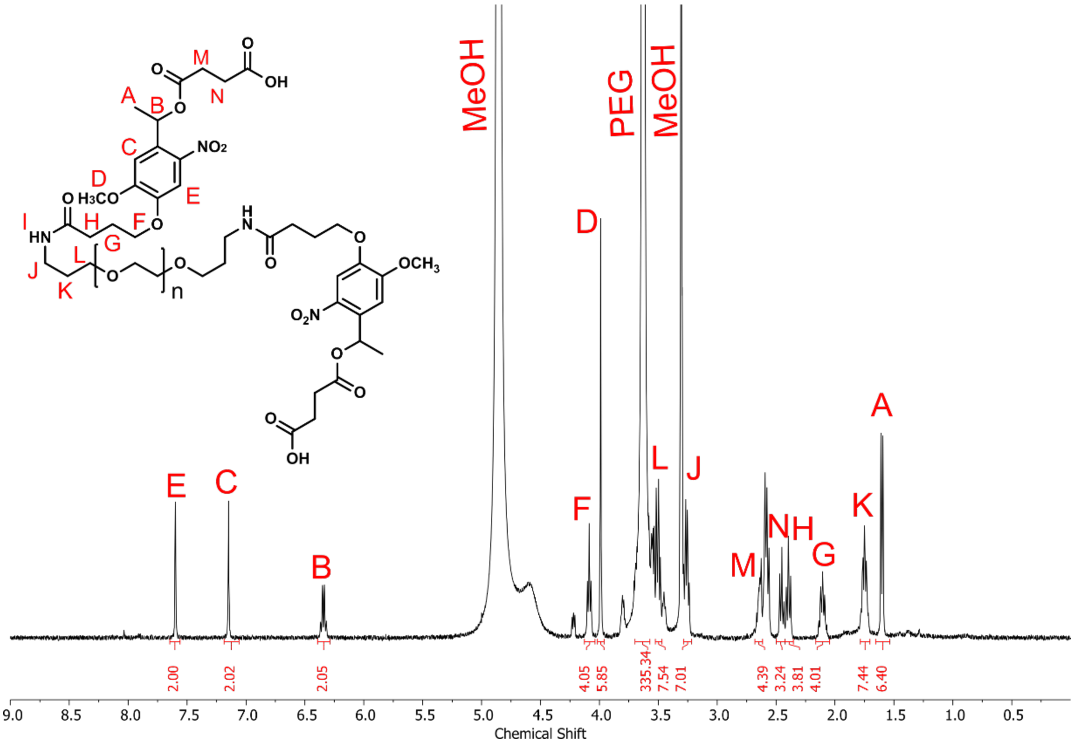

Supplementary Figure S11 ^{1}H NMR spectrum of **(3)** recorded on 400 MHz NMR spectrometer in CD_3OD and calibrated against residual methanol.

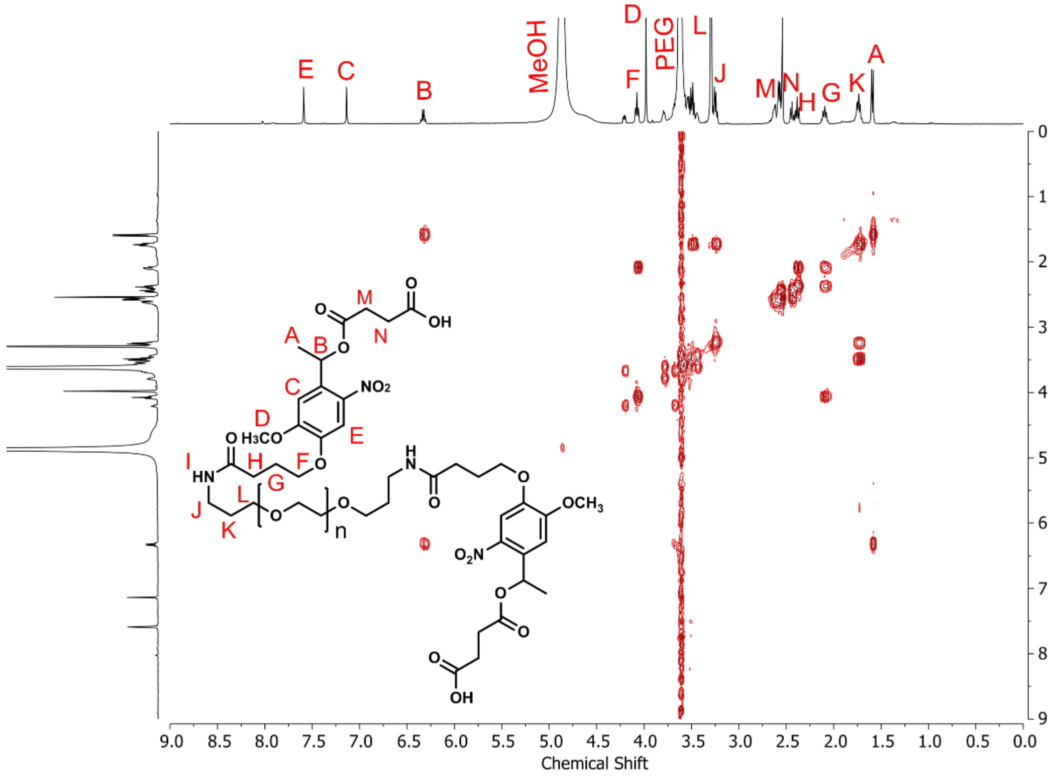

Supplementary Figure S12 2D ^{1}H COSY NMR spectrum of **(3)** recorded on a 400 MHz NMR spectrometer in CD_3OD and calibrated against residual methanol.

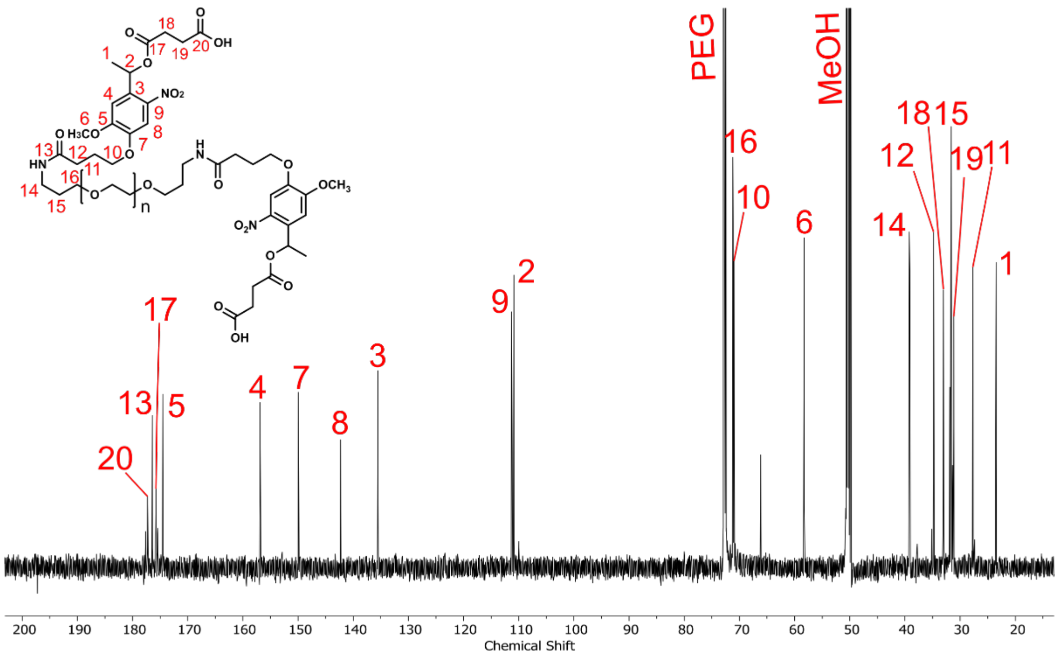

Supplementary Figure S13 ^{13}C NMR spectrum of **(3)** recorded on 500 MHz NMR spectrometer in CD_3OD and calibrated against methanol.

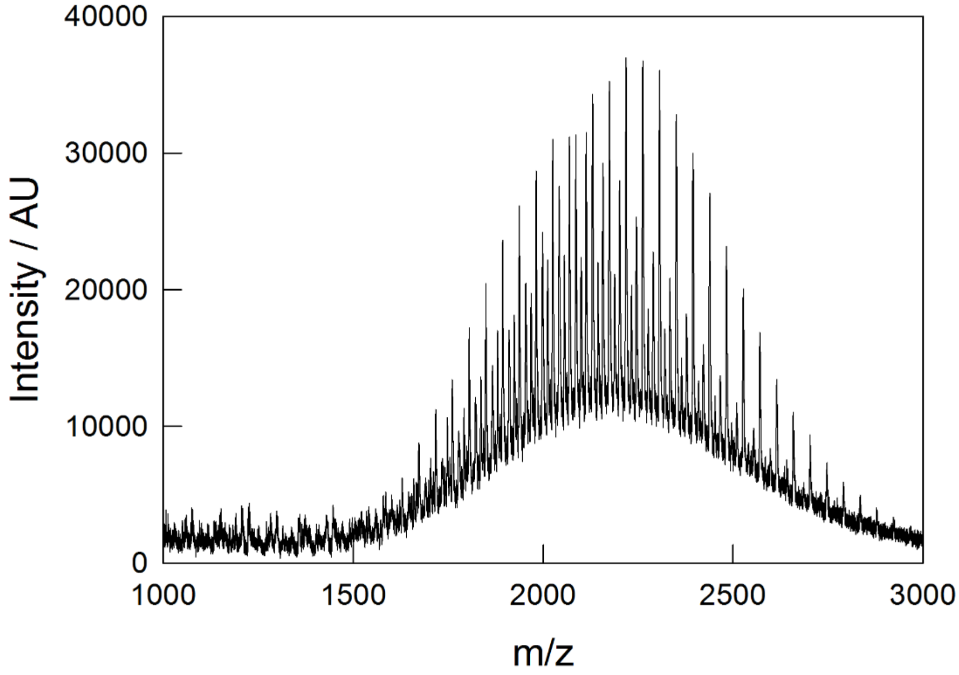

Supplementary Figure S14 MALDI-TOF spectrum of **(3)** recorded using alpha-cyano-4-hydroxycinnamic acid (7 mg/mL in ACN/0.1%TFA) as the matrix solution.

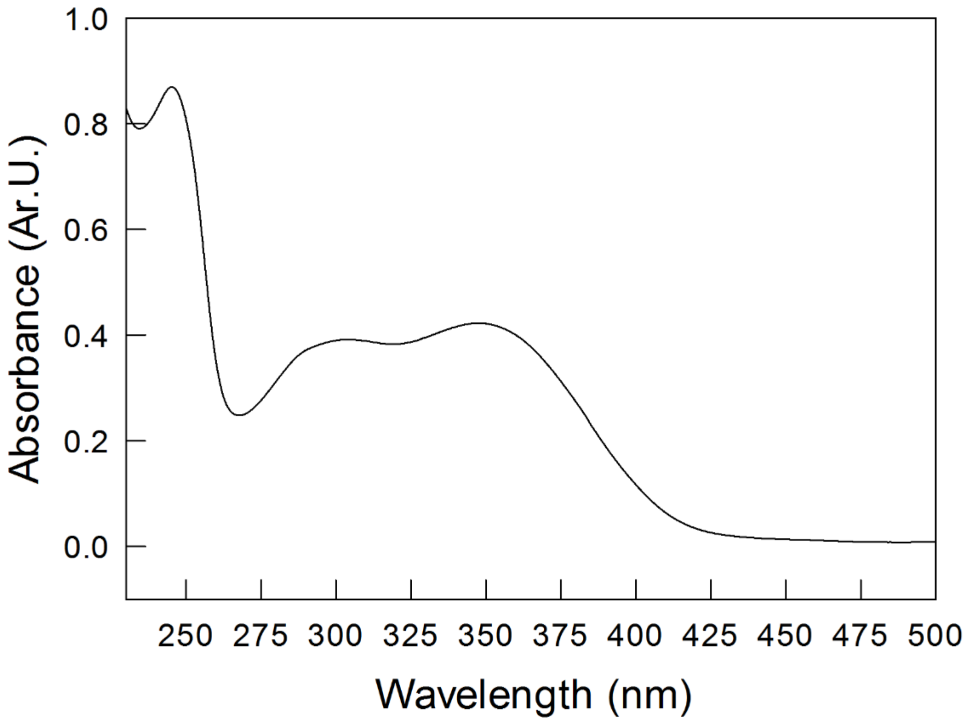

Supplementary Figure S15 UV-Vis spectrum of a 0.1 mM solution of **(3)** in PBS buffer (0.1 M, pH 7.4).

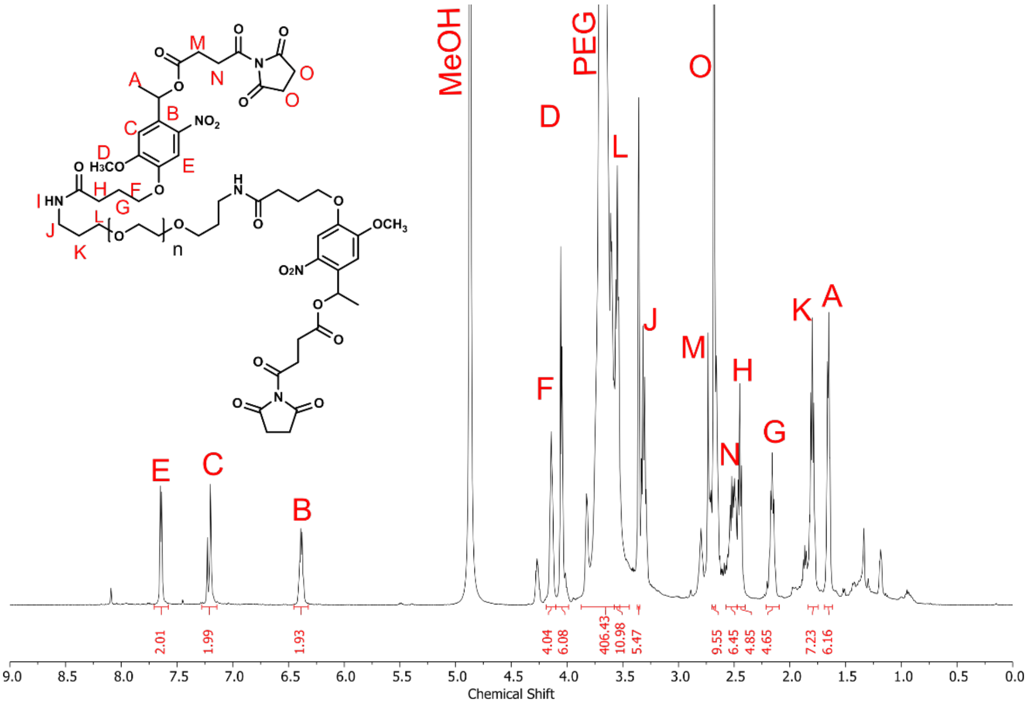

Supplementary Figure S16 ^{1}H NMR spectrum of **(4)** recorded on 500 MHz NMR spectrometer in CD_3OD and calibrated against residual methanol.

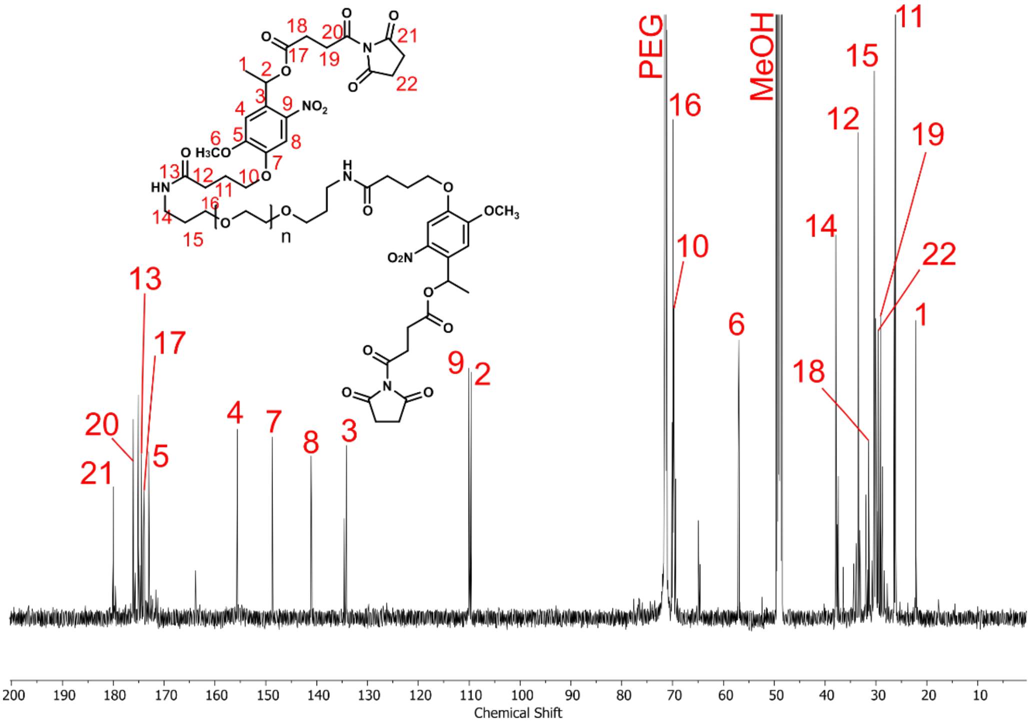

Supplementary Figure S17 ^{13}C NMR spectrum of **(4)** recorded on 500 MHz NMR spectrometer in CD_3OD and calibrated against residual methanol.

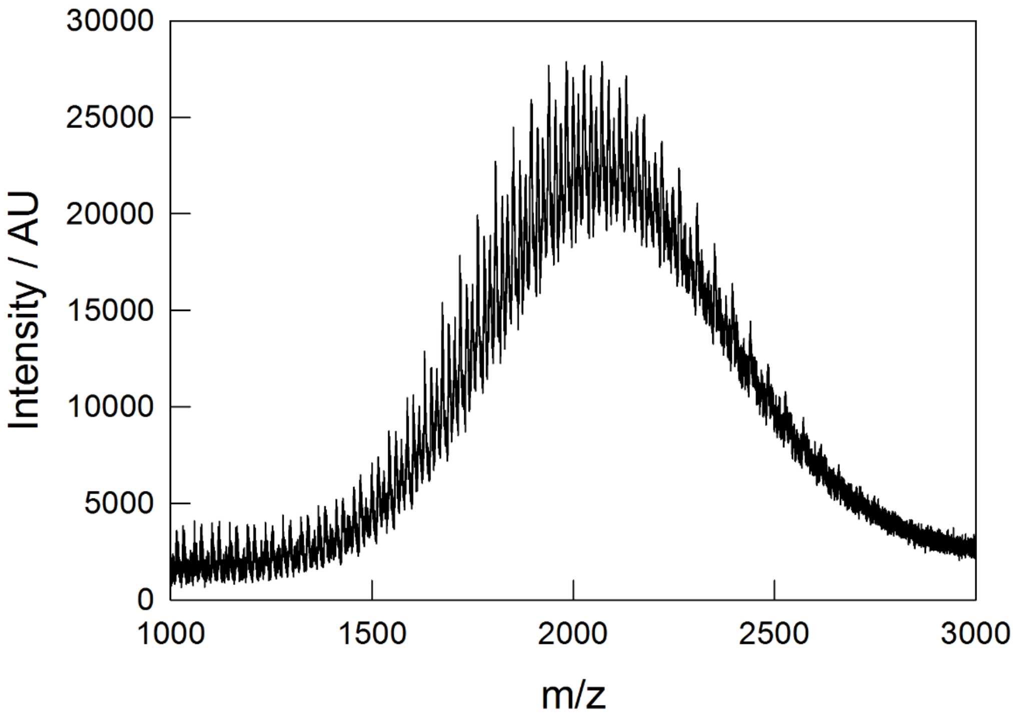

Supplementary Figure S18 MALDI-TOF spectrum of **(4)** recorded using alpha-cyano-4-hydroxycinnamic acid (7 mg mL^{-1} in ACN/0.1%TFA) as the matrix solution.

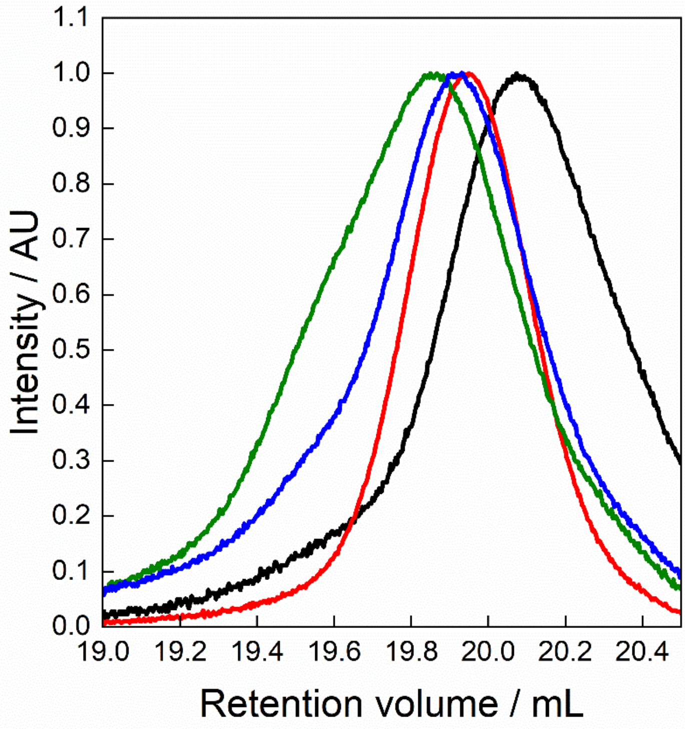

Supplementary Figure S19 Gel permeation chromatograph of the PEG-diNH_2 starting material (black curve), compared to compounds **(2)** (red curve), **(3)** (green curve), and **(4)** (blue curve).

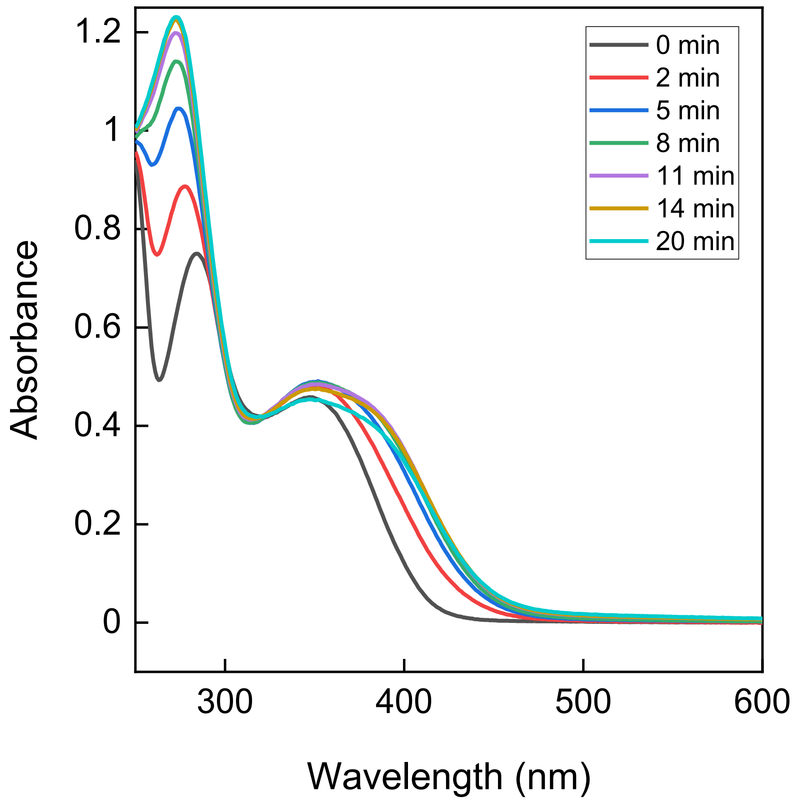

Supplementary Figure S20 UV-Vis kinetics of photoreaction of 0.1 mM solution of **(3)** in phosphate buffered saline (100 mM, pH 7.4). The solution was irradiated for 20 minutes with a 365 nm xenon light source (11 mW) individual spectra being recorded at the time point shown in the legend of the figure.

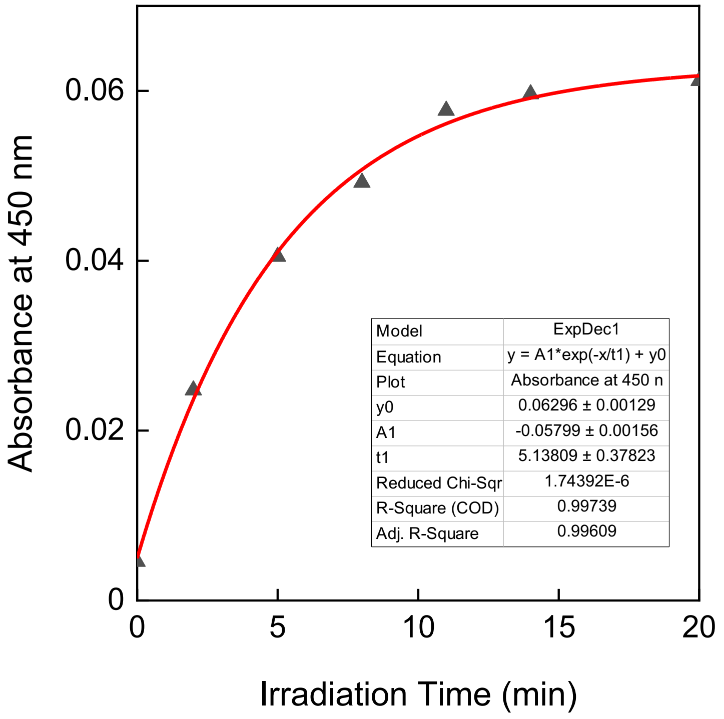

Supplementary Figure S21 Data points of absorbance at 450 nm (from figure S20) plotted against irradiation time and fitted with exponential decay function.

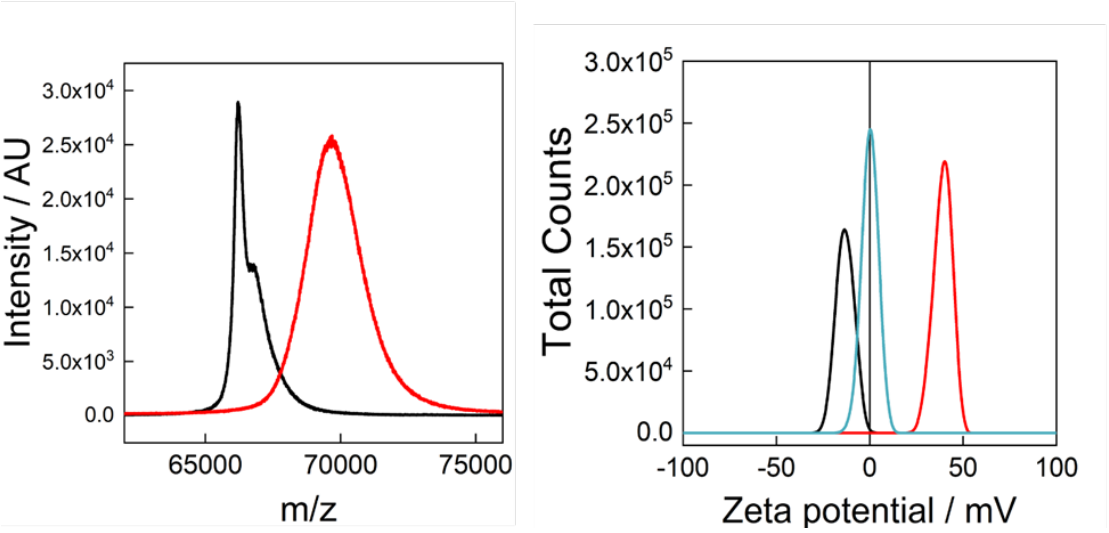

Supplementary Figure S22 MALDI-TOF spectra (left graph) and Zeta potential (right graph) measurements of RITC-labelled BSA (black plots) and cationised RITC-labelled BSA (red plots). The zeta potential of RITC-labelled BSA/PNIPAAm nanoconjugate is also shown on the right graph (blue plot).

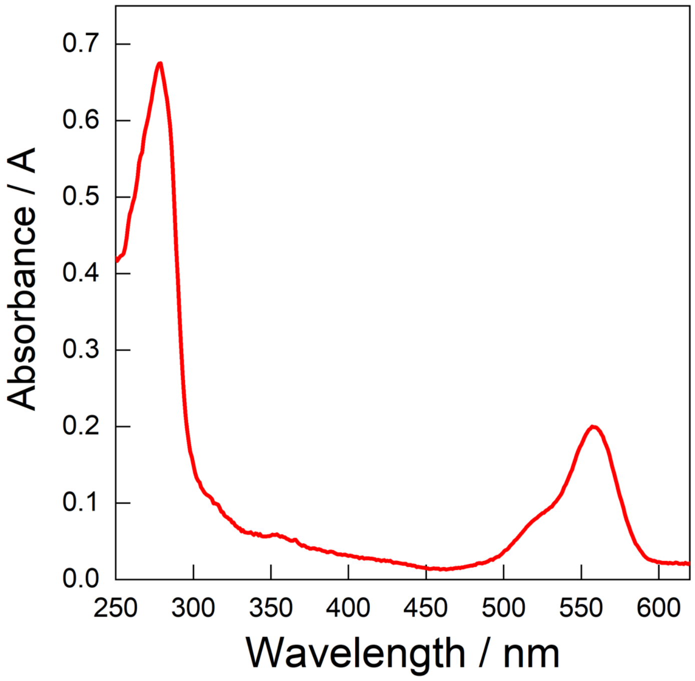

Supplementary Figure S23 UV-Vis spectrum of a 1 mg mL^{-1} solution of RITC-tagged BSA in water. The peak at approx. 280 nm corresponds to absorbance of the protein and the peak at approx. 550 nm corresponds to the absorbance of RITC.

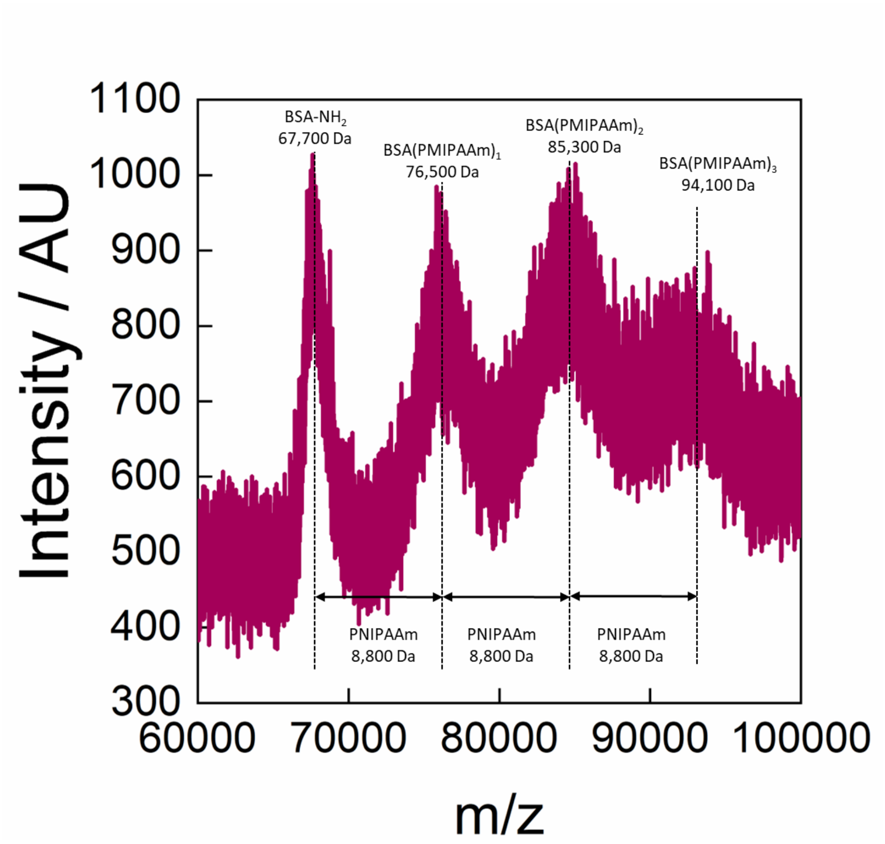

Supplementary Figure S24 MALDI-TOF spectrum of RITC-labelled BSA/PNIPAAm nanoconjugate showing the addition of 0, 1, 2 and 3 PNIPAAm (M_n = approx. 8,800 Da) chains onto the BSA protein

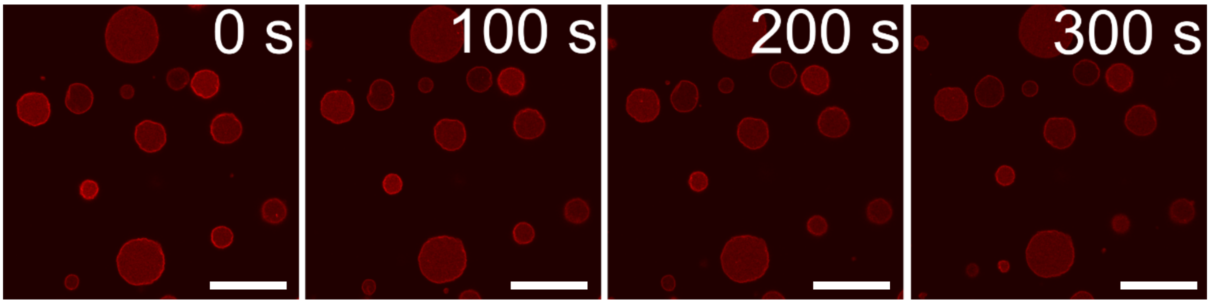

Supplementary Figure S25 Control experiment with proteinosomes crosslinked with PEG-diNHS during 300 seconds of irradiation with a 50 mW 405 laser. Scale bars = 50 µm.

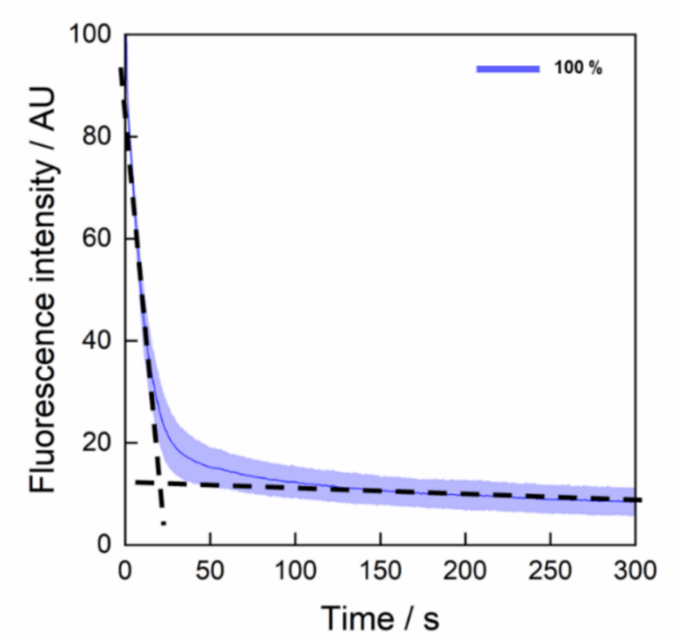

Supplementary Figure S261 Time-dependent fluorescence intensity decay for a photo-sensitive proteinosome irradiated with 405 nm light. Data fitted with two linear regressions (black dashed lines) to define a "proteinosome degradation time", defined as the time value at the intercept of the two linear regressions.

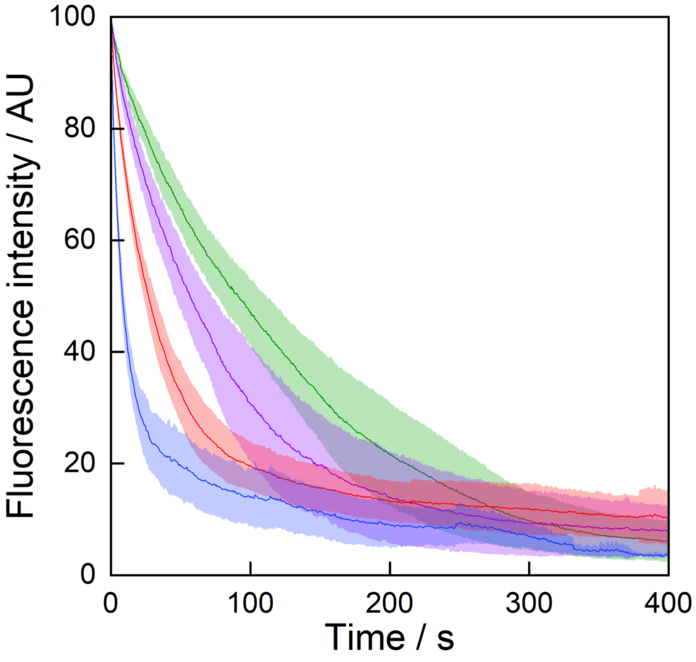

Supplementary Figure S27 Time-dependent fluorescence intensity decay for an RITC-labelled *o*-NB proteinosome irradiated with 405 nm light at different laser scan speeds (0.63 μm s^{-1} = blue line, 0.49 μm s^{-1} = red line, 0.33 μm s^{-1} = purple line, 0.16 μm s^{-1} = green line). Bands = standard deviation.

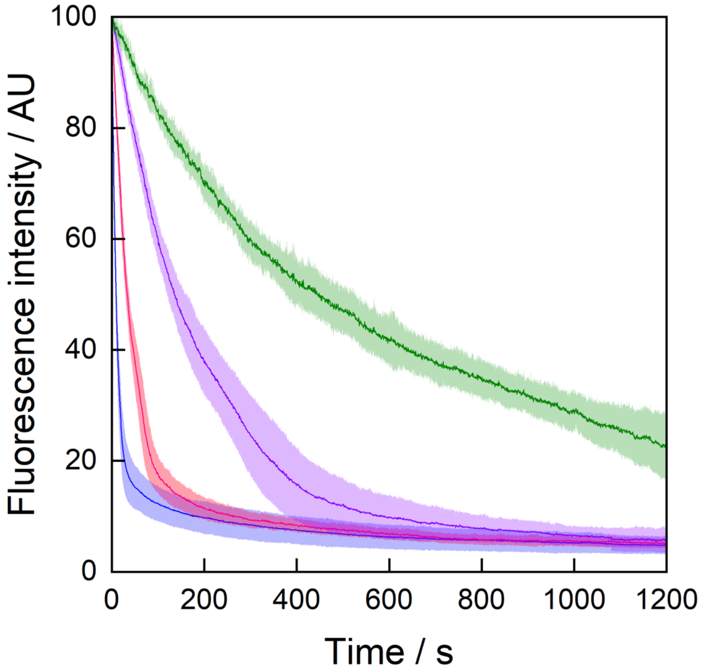

Supplementary Figure S28 Time-dependent fluorescence intensity decay for an RITC-labelled *o*-NB proteinosome irradiate with 405 nm light with different laser powers (100 % = blue line, 25 % = red line, 10 % = purple line, 5 % = green line). Bands = standard deviation. The initial rate of disassembly increases from 0.6 to 7.2 % s^{-1} for 10 to 100 % laser power, respectively.

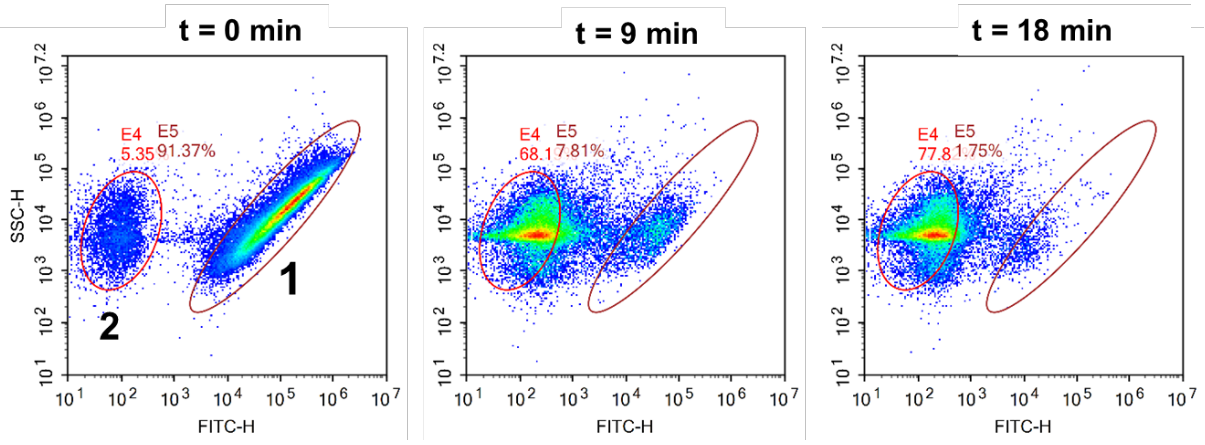

Supplementary Figure S29 2D plots of fluorescence intensity (FITC-H) vs. side scattered light area (SSC-A) for proteinosomes crosslinked with PEG-o-NB-NHS. Population **1** with higher fluorescence is made up of single proteinosomes. Population **2** was hypothesised to be proteinosome membrane fragments and contaminants formed as a result of transfer of the proteinosomes from bulk oil to bulk aqueous phase. As irradiation time increases from 0 to 9 to 18 min, the bulk of the signal moves from the region of population **1** to population **2**, indicating light-induced proteinosome degradation.

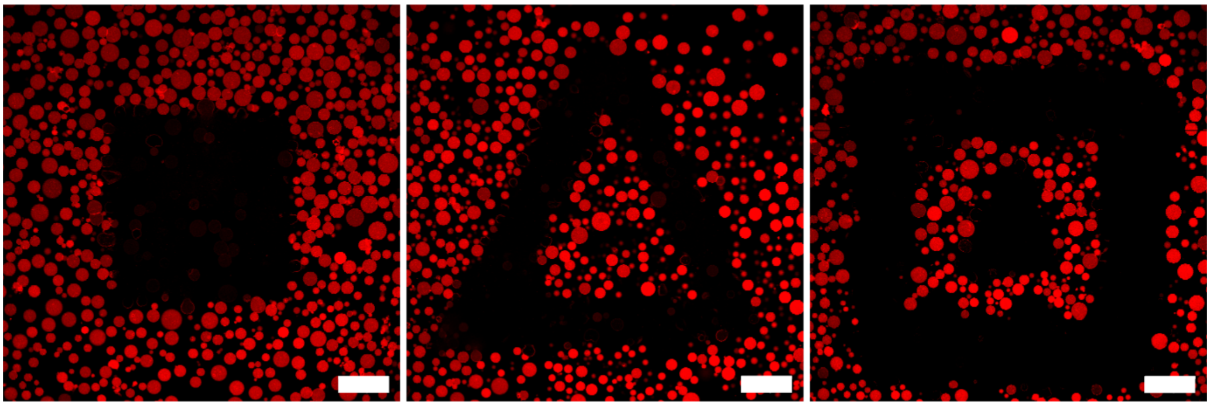

Supplementary Figure S30 Photo-generated proteinosome patterns obtained by irradiating a sample of RITC-labelled photo-sensitive proteinosomes for 5-10 mins at 405 nm using a CLSM equipped with a software-controlled positioning system. These experiments show that it is possible to photo-generate more complex patterns within a population of proteinosomes very easily. For example, the latter two images which show how positive and negative shapes can be embedded within one another. All scale bars = 100 μm.

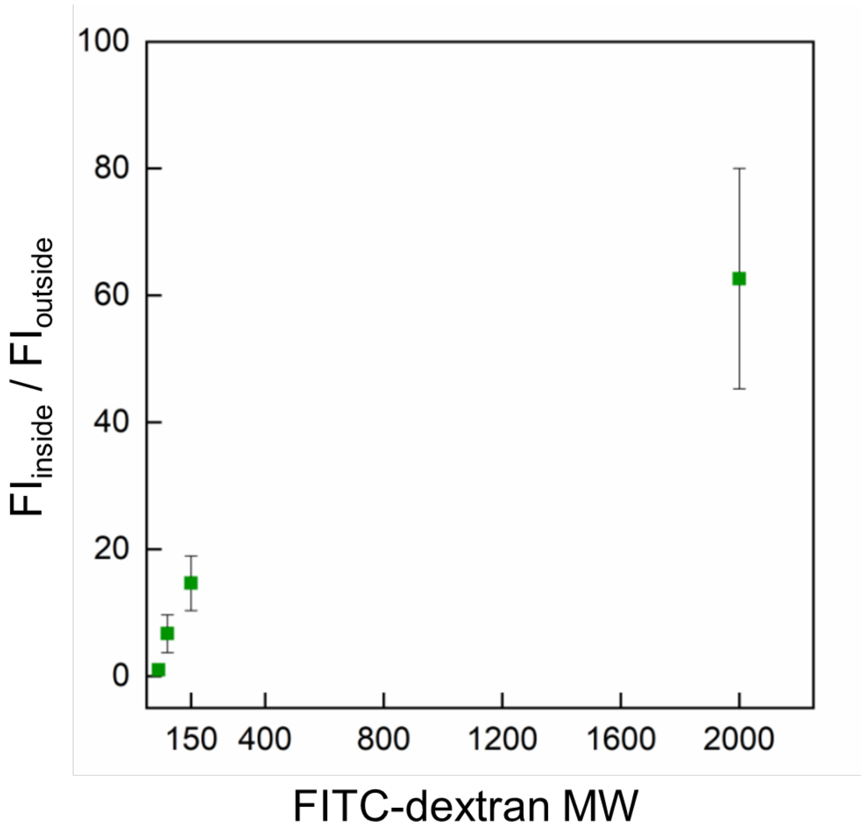

Supplementary Figure S312 Graph describing the ratio of fluorescence intensity inside the proteinosome membrane (FI_{inside}) to outside the proteinosome membrane ($FI_{outside}$) against molecular weight (MW) of FITC-dextran.

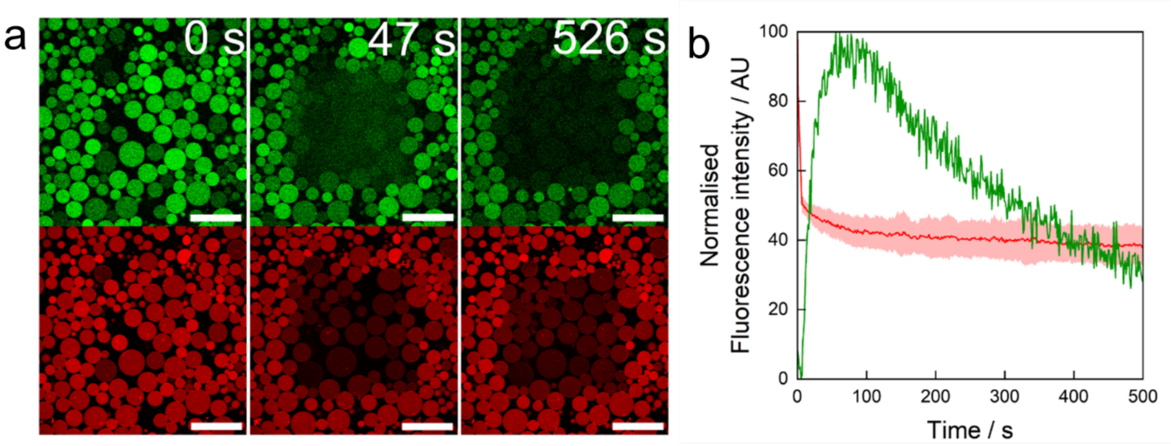

Supplementary Figure S32 Selective cargo release from photo-sensitive proteinosomes. **a**, Time-dependent CLSM images of RITC-labelled photo-sensitive proteinosomes containing FITC-labelled Dextran (150 kDa, 6.67 μM final concentration) irradiated for 5.5 sec (laser wavelength: 405 nm; laser power: 50 mW) starting at 0s. The green channel (top) shows the progressive release of the Dextran cargo from the lumen of the irradiated proteinosomes; the red channel (bottom) shows instead that after the initial photo-generation of membrane pores, the photo-sensitive proteinosomes remain stable. The times reported on top of each pair of images indicate the time elapsed from the beginning of the experiment. Scale bars = 100 μm. **b**, Graph reporting the time-dependent changes in the fluorescence intensity of the RITC-labelled photo-sensitive proteinosome (red line) and of the background fluorescence (green line) for the experiment in (**a**). Band = standard deviation.

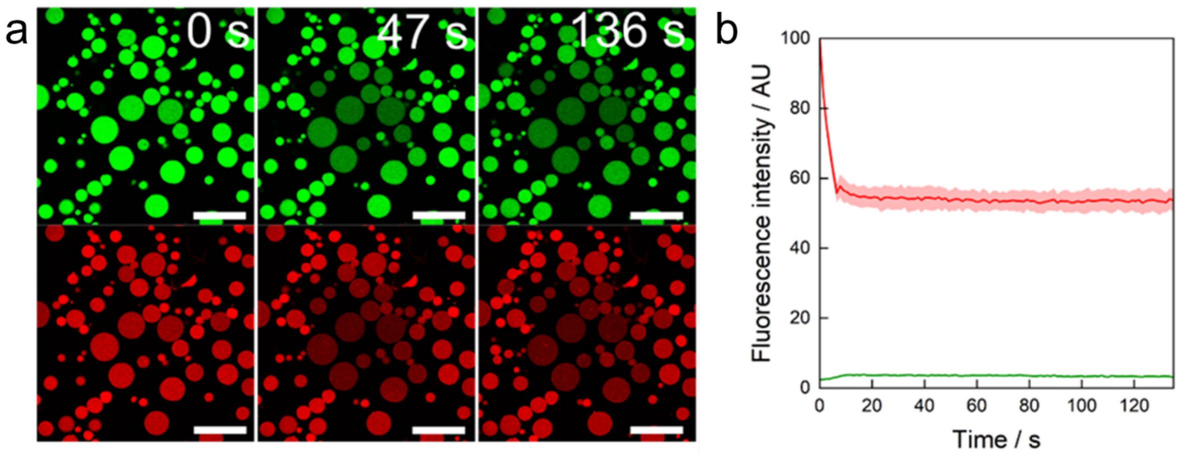

Supplementary Figure S33 Selective **c**argo release from photo-sensitive proteinosomes. **a**, Time-dependent CLSM images of RITC-labelled photo-sensitive proteinosomes containing FITC-labelled Dextran (2000 kDa, 6.67 μM final concentration) irradiated for 5.5 sec (laser wavelength: 405 nm; laser power: 50 mW) starting at 0s. The green channel (top) shows that the high molecular weight Dextran cargo remains caged inside the lumen of the irradiated proteinosomes because the photo-generated pores are not large enough to favour the release of the polymer; the red channel (bottom) shows that after the initial photo-generation of membrane pores, the photo-sensitive proteinosomes remain stable. The times reported on top of each pair of images indicate the time elapsed from the beginning of the experiment. Scale bars = 100 μm. **b**, Graph reporting the time-dependent changes in the fluorescence intensity of the RITC-labelled photo-sensitive proteinosome (red plot) and of the background fluorescence (green plot) for the experiment in (**a**). Band = standard deviation.

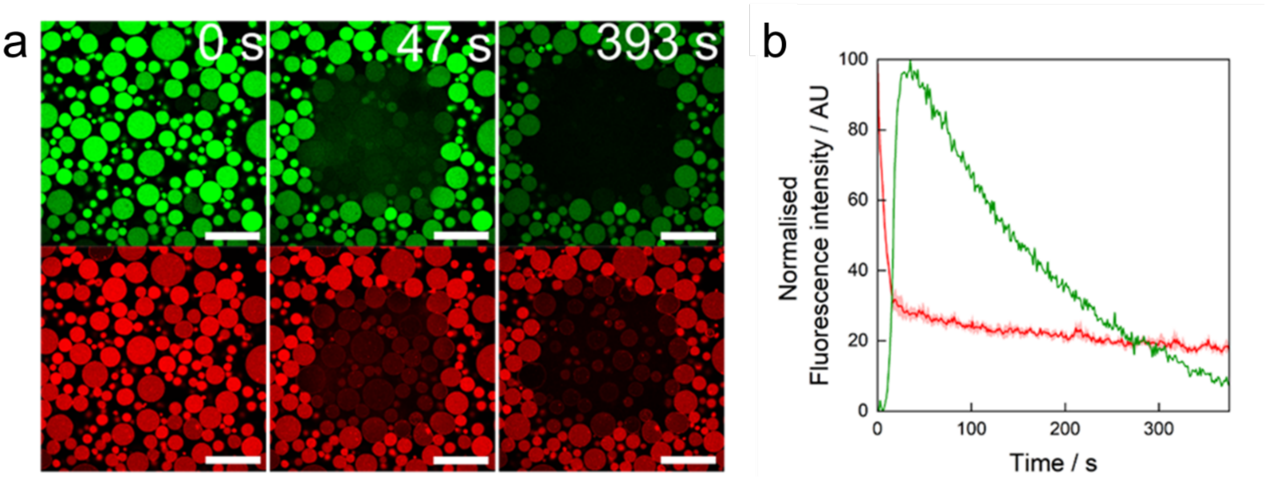

Supplementary Figure S34 Selective cargo release from photo-sensitive proteinosomes. **a**, Time-dependent confocal laser scanning microscopy images of RITC-labelled photo-sensitive proteinosomes containing FITC-labelled Dextran (2000 kDa, 6.67 µM final concentration) irradiated for 15.5 sec (laser wavelength: 405 nm; laser power: 50 mW) starting at 0s. The green channel (top) shows the progressive release of the Dextran cargo from the lumen of the irradiated proteinosomes; the red channel (bottom) shows instead that after the initial photo-generation of membrane pores, the photo-sensitive proteinosomes remain stable. The times reported on top of each pair of images indicate the time elapsed from the beginning of the experiment. Scale bars = 100 µm. **b**, Graph reporting the time-dependent changes in the fluorescence intensity of the RITC-labelled photo-sensitive proteinosome (red plot) and of the background fluorescence (green plot) for the experiment in (**a**). Band = standard deviation.

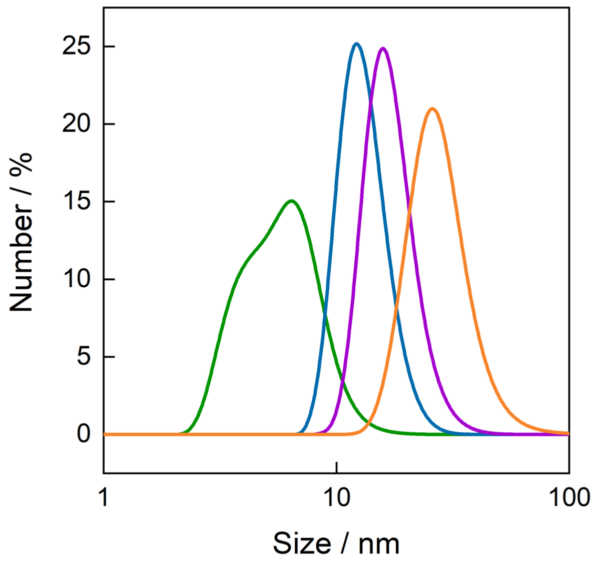

Supplementary Figure S35 Plots of size of FITC-dextran determined using DLS. Hydrodynamic radii of 40 (green curve), 70 (blue curve), 150 (purple) and 2000 (orange curve) kDa FITC-dextran were found to be 5.6 ± 1.6 nm, 12.3 ± 0.9 nm, 16.5 ± 1.2 nm and 20.1 ± 1.4 nm respectively.

4. Supplementary Videos

Supplementary Video 1: **Photo-induced cleavage of PEG-*o*-NB-NHS crosslinked proteinosomes on irradiation with 405 nm laser.** Fluorescence CLSM video of PEG-*o*-NB-NHS crosslinked proteinosomes in water, synthesised from BSA/PNIPAAm nanoconjugates tagged with RITC. The CLSM positioning software was used to only irradiate a central portion of the frame with the 405 nm laser, whilst the entirety of the frame was exposed to the 576 nm laser to visualise the RITC proteinosome population. Proteinosome membranes in the central portion of the video frame are shown to be cleaved during exposure to the UV laser for a period of 3 minutes. Only those proteinosomes which entered the region being irradiated with the 405 nm laser were cleaved.

Supplementary Video 2: Selective photo-induced disassembly of a binary population of photo-sensitive and control proteinosomes. Fluorescence CLSM video showing two proteinosomes, a photo-sensitive, FITC-tagged, PEG-*o*-NB-NHS crosslinked proteinosome (green) and a photo-stable, RITC-tagged, PEG-diNHS crosslinked proteinosome (red). Both proteinosomes were simultaneously irradiated with a 405 nm laser (50 mW) over a period of 72 s, inducing photolysis only in the green proteinosome.

Supplementary Video 3: Fluorescence CLSM video showing a mixed population of proteinosomes: photo-sensitive, FITC-tagged, PEG-*o*-NB-NHS crosslinked proteinosomes (green) and photo-stable, RITC-tagged, PEGdiNHS crosslinked proteinosomes (red). The mixed population of proteinosomes was simultaneously irradiated with a 405 nm laser (50 mW) over a period of 128 s, inducing photolysis only in the green proteinosomes.

5. Supplementary References

[1] I. Murgorodska, M. Jenkinson-Finch, R. O. Moreno-Tortolero, S. Mann*, P. Gobbo*, *Macromol. Rapid Commun.*, **2021**, *42*, 2100102.
[2] P. G. d. Gennes, T. A. Witten, *Physics Today* **1980**, *33*, 51-54.
[3] D. Chithrani, *Journal of Nanomedicine Research* **2014**, *1*, 27-32.
